# HPD is an RNA‐Binding Protein Sustaining Ovarian Cancer Cell Glycolysis, Tumor Growth, and Drug Resistance

**DOI:** 10.1002/advs.202503999

**Published:** 2025-06-10

**Authors:** Fei Xie, Han Zhang, Xintong Dai, Mengxin Tu, Chenxi Yu, Lumeng Liu, Yajuan Guo, Huanran Sun, Qingle Gao, Jiyan Wang, Mingming Sun, Qijun Zhang, Taoyuan Wang, Tao He, Zhen Li, Yanping Li, Tao Wang, Jianguo Zhao, Zhongjie Chen, Chunze Zhang, Shuai Zhang, Changliang Shan

**Affiliations:** ^1^ State Key Laboratory of Medicinal Chemical Biology College of Pharmacy and Tianjin Key Laboratory of Molecular Drug Research Nankai University Tianjin 300350 China; ^2^ Department of Pharmacology School of Pharmacy Fourth Military Medical University Xi'an 710032 China; ^3^ Cardiothoracic Surgery Department Characteristic Medical Center of the Chinese People's Armed Police Force Tianjin 300162 China; ^4^ Department of Pathology Characteristic Medical Center of The Chinese People's Armed Police Force Tianjin 300162 China; ^5^ Guangzhou Key Laboratory for Clinical Rapid Diagnosis and Early Warning of Infectious Diseases KingMed School of Laboratory Medicine Guangzhou Medical University Guangzhou Guangdong 510180 China; ^6^ Department of Pathology and Institute of Precision Medicine Jining Medical University Jining 272067 China; ^7^ Tianjin Key Laboratory of Human Development and Reproductive Regulation Tianjin Central Hospital of Obstetrics and Gynecology Tianjin 300110 China; ^8^ Department of Radiation Oncology, Tianjin Medical University Cancer Institute and Hospital National Clinical Research Center for Cancer Key Laboratory of Cancer Prevention and Therapy Tianjin's Clinical Research Center for Cancer Tianjin 300060 China; ^9^ Department of Radiation Oncology Tianjin Cancer Hospital Airport Hospital Tianjin 300308 China; ^10^ Department of Colorectal Surgery Tianjin Union Medical Center and The First Affiliated Hospital of Nankai University Tianjin 300121 China; ^11^ School of Integrative Medicine Tianjin University of Traditional Chinese Medicine Tianjin 301617 China

**Keywords:** 4‐hydroxyphenylpyruvate dioxygenase (HPD), glycolysis, mRNA translation, ovarian cancer, RNA‐binding protein

## Abstract

4‐hydroxyphenylpyruvate dioxygenase (HPD) is an important metabolic enzyme in the tyrosine metabolic pathway and displays aberrant expression and function in cancer. Unexpectedly, it is discovered that HPD functions as an RNA‐binding protein (RBP) to drive ovarian cancer progression. HPD is shown to bind to the RRACH motif of these target mRNAs through its two dsRNA binding domains (RBDs), resulting in increased global mRNA translation. In particular, HPD binding is demonstrated to mediate translation of glycolytic enzymes triosephosphate isomerase (TPI) and alpha‐enolase (ENO1) mRNAs, which facilitates ovarian cancer glycolysis and tumor growth. Thus, targeting the RBD domain of HPD disrupts its RNA binding ability, leading to blocking glycolysis flux, tumor growth, and enhancing drug response. HPD is a novel RNA‐binding protein, and this moonlighting function highlights the knowledge of HPD in regulating cancer development and drug response beyond only as a metabolic enzyme.

## Graphical abstract

HPD is identified as an RNA‐binding protein that promotes mRNA translation by binding to RRACH motifs via its double‐stranded RNA‐binding domains. This RNA‐binding activity critically sustains glycolysis in ovarian cancer cells. Disrupting HPD's RNA‐binding function effectively suppresses tumor growth and enhances therapeutic sensitivity, highlighting its potential as a novel target for ovarian cancer treatment.

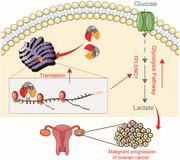

## 1 Introduction

Most tumor cells exhibit increased glucose uptake and glycolysis under aerobic conditions, a phenomenon known as the Warburg effect or aerobic glycolysis. They use anaerobic glycolysis as a low‐energy and efficient way to ensure the survival of tumor cells and promote the proliferation of tumor cells even under aerobic conditions.^[^ ^1^ ^]^ Interestingly, recent studies have found that several metabolic enzymes have moonlighting functions, which are involved in the regulation of cell cycle, DNA damage repair, cell proliferation, survival, apoptosis pathway, and tumor microenvironment, thus affecting the occurrence and progression of tumors and therapeutic efficacy.^[^ ^2^, ^3^ ^]^ For example, phosphoglycerate kinase 1 (PGK1) undergoes mitochondrial translocation under hypoxic conditions, which phosphorylates pyruvate dehydrogenase kinase 1 (PDHK1), reduces mitochondrial pyruvate utilization, and promotes tumorigenesis.^[^ ^4^ ^]^ Therefore, an in‐depth understanding of the moonlighting functions of metabolic enzymes will deepen the understanding of the field of tumor metabolism and provide potential therapeutic targets and new strategic ideas for tumor clinical practice.

4‐hydroxyphenylpyruvate dioxygenase (HPD) is the second key enzyme in the tyrosine catabolism pathway, which catalyzes the conversion of 4‐hydroxyphenylpyruvic acid (4‐HPPA) to homogentisic acid (HGA).^[^ ^5^ ^]^ Recent discoveries show that HPD displays aberrant expression and function in breast, lung, and liver cancer.^[^ ^6^, ^7^ ^]^ We found that HPD promotes lung cancer growth mediated by activating the AMPK‐HDAC10‐G6PD axis, leading to enhanced pentose phosphate flux.^[^ ^8^ ^]^ Tong et al found that the absence of HPD in liver cancer promotes glutamine dependence and supports liver cancer, suggesting that HPD may be used as a metabolic vulnerability for liver cancer treatment.^[^ ^9^ ^]^ However, whether HPD has a moonlighting function in regulating cancer development and drug response has not been reported. Interestingly, it has been found that 4‐hydroxyphenylpyruvate dioxygenase‐like protein (HPDL), the homologous protein of HPD, is a potential RNA‐binding protein.^[^ ^10^ ^]^ This further inspired us to explore the moonlighting function of HPD. In this study, we made the striking discovery that HPD is a non‐canonical RNA‐binding protein (RBP) with a previously unknown function as a regulator of mRNA translation.

RNA‐binding proteins (RBPs) are a very important class of proteins in cells.^[^ ^11^ ^]^ They are widely involved in multiple post‐transcriptional regulatory processes such as RNA splicing, transport, sequence editing, intracellular localization, and translation control through special RNA binding domains (such as RNA recognition motifs, K homology domains, double‐stranded RNA binding domains, zinc finger domains, etc.) that bind to specific motifs on RNA or RNA secondary structures^[^ ^6^, ^7^, ^12^ ^]^ Their ability to bind RNA serves as powerful and extensive regulatory factors, which play an important role in cell development, differentiation, metabolism and a variety of diseases.^[^ ^11^, ^13^ ^]^ Recent reports show that the non‐canonical RBPs have expanded the broader category of RNA‐binding proteins. For example, ERa is a non‐canonical RBP sustaining tumor cell survival and drug resistance as a regulator of RNA metabolism.^[^ ^14^ ^]^ In particular, several studies have shown that metabolic enzymes also play a regulatory role as RBP.^[^ ^15^ ^]^ For example, alpha‐enolase (ENO1) serves as a non‐canonical RBPs to regulate iron homeostasis and survival of hepatocellular carcinoma (HCC) cells by suppressing iron regulatory protein 1 (IRP1) expression.^[^ ^16^ ^]^ In addition, ENO1 promotes liver carcinogenesis through Yes‐associated protein1 (YAP1)‐dependent arachidonic acid metabolism by promoting YAP1 translation.^[^ ^17^ ^]^ There are many proteins known to regulate cancer development and drug response. There are also other non‐canonical RBPs known to regulate cancer.^[^ ^18^ ^]^ However, it is still unknown whether other metabolic enzymes play a role in regulating cancer development and drug response as non‐canonical RBPs, such as HPD.

In the current study, we surprisingly demonstrate that HPD works as an RBP to promote ovarian cancer glycolysis flux by increasing global mRNA translation, including glycolytic metabolic enzymes TPI and ENO1, thereby promoting the tumorigenesis of ovarian cancer. Furthermore, we uncover the underlying mechanism of HPD as an RNA‐binding protein, which binds to the RRACH motif of mRNA through its own two dsRNA binding domains. Thus, blocking the RBD domain of HPD destroys its RNA‐binding function, thereby inhibiting global mRNA translation, leading to the inhibition of cancer development. Collectively, our results demonstrate that the metabolic enzyme HPD acts as a non‐canonical RBP to promote global mRNA translation efficiency in ovarian cancer, adding another dimension to the regulation of ovarian cancer development of HPD with moonlighting function.

## 2 Results

### 2.1 HPD is a New Non‐Canonical RBP and Associates with Multiple mRNAs

**Figure 1:**
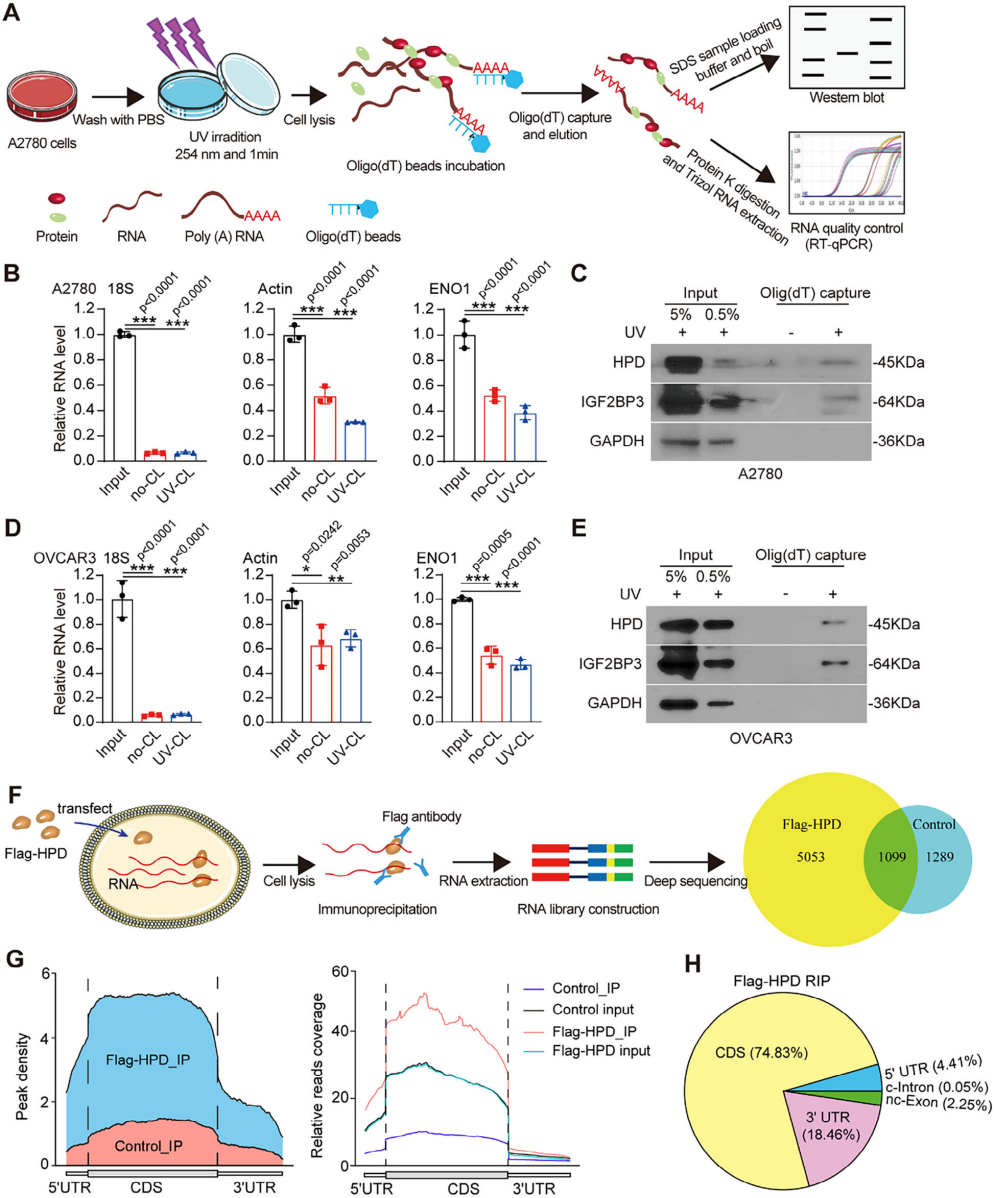
HPD is a new non‐canonical RBP and associates with multiple mRNAs. A) Illustration of the experimental setup to identify the mRNA‐bound proteome. B) The levels of 18S rRNA, β‐actin, and ENO1 in eluates of A2780 cells were evaluated by qPCR assay. (no‐CL, no UV‐crosslinked; UV‐CL, UV‐crosslinked, *n* = 3). C) The levels of HPD, IGF2BP3, and GAPDH in eluates from A2780 cells were analyzed by western blotting. (UV, ultraviolet). D) The levels of 18S rRNA, β‐actin, and ENO1 in eluates of OVCAR3 cells were evaluated by qPCR assay. (no‐CL, no UV‐crosslinked; UV‐CL, UV‐crosslinked, *n* = 3). E) The levels of HPD, IGF2BP3, and GAPDH in eluates from OVCAR3 cells were analyzed by western blotting. (UV, ultraviolet). F) The target mRNAs bound by HPD were analyzed through RNA‐immunoprecipitation sequencing (RIP‐seq). G,H) Distribution of HPD binding regions in the indicated mRNA regions was shown based on RIP‐seq. (Control, *n* = 1; Flag‐HPD, *n* = 1). Error bars in B and D, mean values ± SD, *p* values were determined by unpaired two‐tailed Student's *t* test of *n* = 3 independent biological experiments. ^*^ *p* < 0.05; ^**^ *p* < 0.01; ^***^ *p* < 0.001.

To explore whether HPD functions as a non‐canonical RNA‐binding protein, we pulled down the complexes of mRNA and protein with oligo (dT)_25_ magnetic beads^[^ ^10^, ^19^, ^20^ ^]^ (**Figure** **1**A). We ensured that polyadenylated transcripts, such as mRNAs of Actin and ENO1 containing poly(A) tail, were enriched in the lysates captured by oligo (dT)_25_ beads, while 18S ribosomal RNA (rRNA) without poly(A) tail was only bound to a very small amount as a negative control. (Figure 1B). And as expected, HPD was detected in the positive samples after oligo (dT)_25_ magnetic beads enrichment (Figure 1C). Meanwhile, the insulin‐like growth factor 2 mRNA‐binding protein 3 (IGF2BP3), a well‐known RNA‐binding protein,^[^ ^21^ ^]^ was present in the UV‐crosslinked samples but absent in non‐UV‐crosslinked controls, and glyceraldehyde 3‐phosphate dehydrogenase (GAPDH) as a negative control^[^ ^19^ ^]^ was not captured (Figure 1C). In addition, we also obtained the same results in OVCAR3 cells (Figure 1D,E). To further explore the interaction between mRNA and HPD in the cells, we determined the location of HPD, and found that HPD was located in both the nucleus and cytoplasm (Figure S1A,B, Supporting Information). Interestingly, HPD was co‐localized with nuclear speckles in the nucleus (Figure S1C, Supporting Information). While, HPD was also co‐localized with endoplasmic reticulum in the cytoplasm (Figure S1D, Supporting Information). Thus, these findings indicate that HPD has the potential to bind RNA in both the nucleus and the cytoplasm.

Lastly, to investigate the RNA targets of HPD across the genome, we performed RNA‐immunoprecipitation sequencing (RIP‐seq), and found that a large number of mRNAs could be bound by HPD (Figure 1F). Kyoto Encyclopedia of Genes and Genomes (KEGG) pathway analysis and Gene Ontology (GO) analysis^[^ ^22^, ^23^ ^]^ on the 5053 RNAs enriched by HPD, which are crucial for cancer progression, including the cell cycle and glycolysis pathways (Figure S1E, Supporting Information). Further analysis of RIP‐seq data revealed that HPD mainly binds to the CDS region of mRNA. (Figure 1G,H; Table S1, Supporting Information). Collectively, our results suggested that HPD is a new non‐canonical RBP and is associated with multiple mRNAs.

### 2.2 The RNA‐Binding Activity of HPD Contributes to Global mRNA Translation

**Figure 2:**
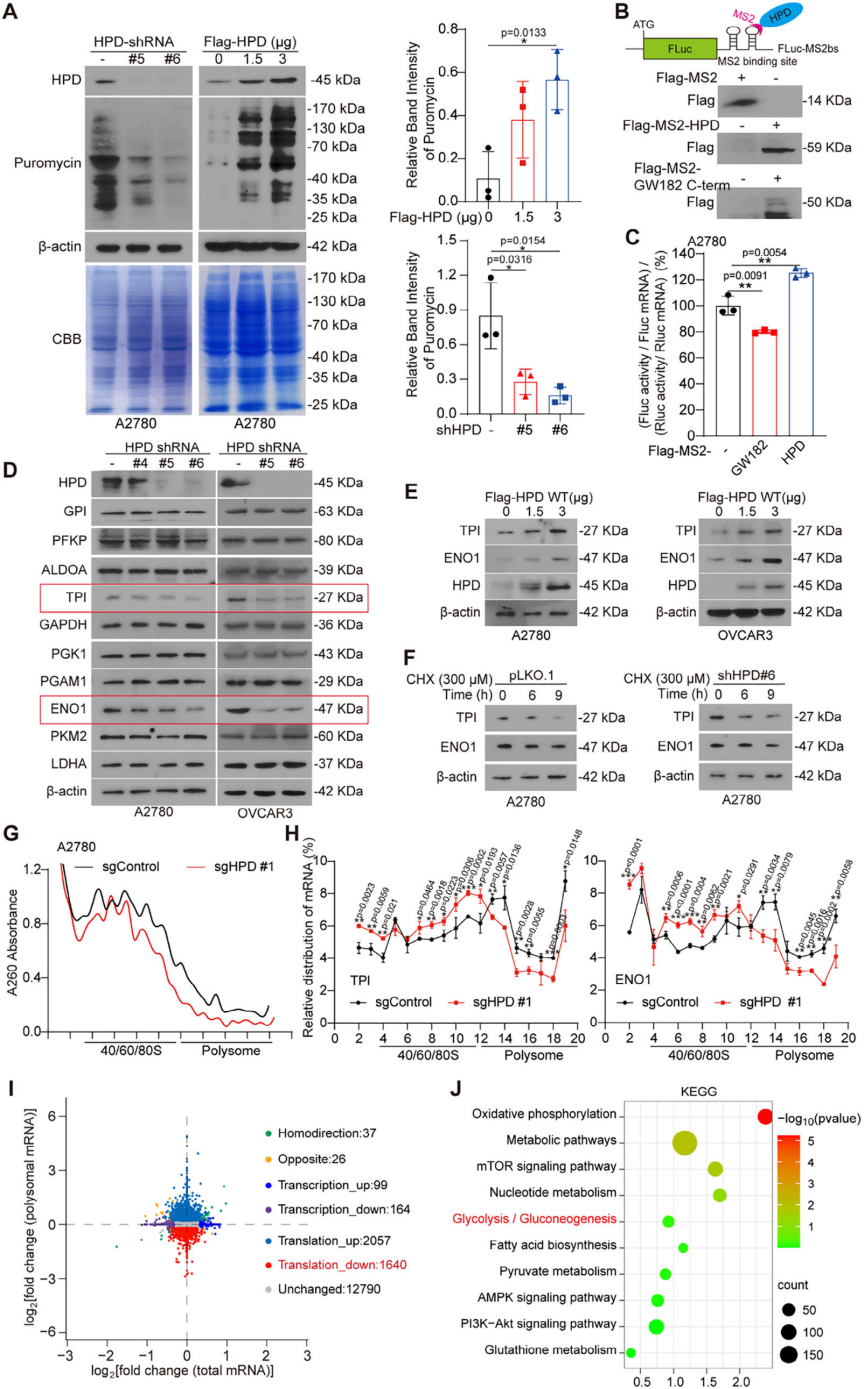
The RNA‐binding activity of HPD contributes to global mRNA translation. A) The puromycylation assay was performed to detect global translation efficiency in A2780 cells with knockdown or exogenous expression of HPD. CBB (Coomassie Brilliant Blue) staining was used to verify the consistency of protein loading amounts. B) *Upper*, Schematic diagram of tethering reporter assay. *Lower*, Western blotting was performed to detect the expression of Flag‐MS2‐HPD/GW182 C‐term. C) Tethering assay was performed to measure translation efficiency of reporter mRNAs. Firefly luciferase (FLuc) activity was measured and normalized to the Renilla luciferase (RLuc) activity. Relative FLuc activity and RLuc activity were normalized to the relative FLuc and Rluc mRNAs, respectively. D) The levels of glycolytic enzymes were detected by western blotting in HPD knockdown A2780 and OVCAR3 cells. E) The levels of TPI and ENO1 were detected by western blotting in A2780 and OVCAR3 cells with exogenous expression of HPD. F) The effect of HPD on the protein stability of TPI and ENO1 was detected by western blotting in the cells treated with CHX. G) Polysome fractionation assay was performed to detect the translation level in A2780 cells with HPD knockout by sucrose density gradient centrifugation. H) Relative mRNA distribution of the TPI and ENO1 in each ribosome fraction from polysome fractionation was analyzed by qPCR assay. I) Ribo‐seq was performed to analyze the polysome‐associated mRNAs, which were regulated by HPD (sgCon, *n* = 3; sgHPD, *n* = 3; Homodirection, up‐regulated and down‐regulated genes in the same direction in both omics; Opposite, up‐regulated and down‐regulated genes in opposite directions in two omics; Transcription_up, up‐regulated genes in RNA‐seq; Transcription_down, down‐regulated genes in RNA‐seq; Translation_up, up‐regulated genes in Ribo‐seq; Translation_down, down‐regulated genes in Ribo‐seq). J) Kyoto Encyclopedia of Genes and Genomes (KEGG) analysis was performed to analyze the down‐regulated genes in the translation level from Ribo‐seq data. Error bars in A, C, and H, mean values ± SD, *p* values were determined by unpaired two‐tailed Student's *t* test of *n* = 3 independent biological experiments. ^*^ *p* < 0.05; ^**^ *p* < 0.01; ^***^ *p* < 0.001.

To determine the regulatory role of HPD on RNA targets, we performed RNA‐seq between HPD knockdown and control cells. Intriguingly, RNA‐seq data showed that more than 80% of the 5053 mRNAs bound to HPD, including multiple glycolytic enzymes, did not change with HPD knockdown. (Figure S2A, Table S2, Supporting Information). Thus, we hypothesize that HPD mainly regulates its translation process after binding to mRNA. To verify this, we carried out puromycylation assay and found that the global translation efficiency increased with the overexpression of HPD and decreased with the knockdown of HPD (**Figure** **2**A). Then, we performed tethering assays (Figure 2B) and observed that Flag‐MS2‐HPD increased the ratio of Fluc activity to Rluc activity (Figure 2C). This data indicated that HPD promotes global mRNA translation as a non‐canonical RBP.

Considering the importance of glycolysis to metabolic reprogramming in cancer, thus, we focus on the mRNA of glycolytic enzymes to explore how HPD promotes global mRNA translation. Then, we found that the protein levels of glycolytic enzymes triose phosphate isomerase (TPI) and enolase (ENO1) were decreased after HPD knockdown (Figure 2D; Figure S2B,C, Supporting Information), while increased with the exogenous expression HPD (Figure 2E). However, the mRNA levels of TPI and ENO1 were not affected by HPD (Figure S2D,E, Supporting Information). Moreover, the mRNA stability of TPI and ENO1 was not regulated by HPD (Figure S2F, Supporting Information). In addition, we also found that knockdown of HPD had no significant effect on the protein stability of TPI and ENO1 (Figure 2F; Figure S2G, Supporting Information). This means HPD regulates the protein levels of target mRNA in a transcription‐independent and protein degradation‐independent manner. These results validate that HPD promotes TPI and ENO1 mRNA translation as a non‐canonical RBP. Furthermore, we performed polysome profiling analysis and found that knocking out HPD decreased the global translation efficiency (Figure 2G). Subsequently, we observed that less mRNA of TPI and ENO1 was accumulated in the polysome fraction of HPD knocked out cells (Figure 2H). Similar results were obtained in A2780 cells that were knocked down HPD (Figure S2H,I, Supporting Information). We next performed Ribo‐seq to confirm the effect of HPD on the translation level. The results showed knockout of HPD caused a decrease in the translation efficiency of 1640 mRNAs, including ENO1 and TPI1 (Figure 2I; Table S3, Supporting Information). Then, we performed KEGG analysis of genes regulated by HPD at the translation level, and found that HPD knockout affected the glycolysis pathway (Figure 2J). These results suggested that HPD acts as a non‐canonical RBP to promote the translation efficiency.

### 2.3 HPD Directly Binds to Targeted mRNA Through RRACH Motif

**Figure 3:**
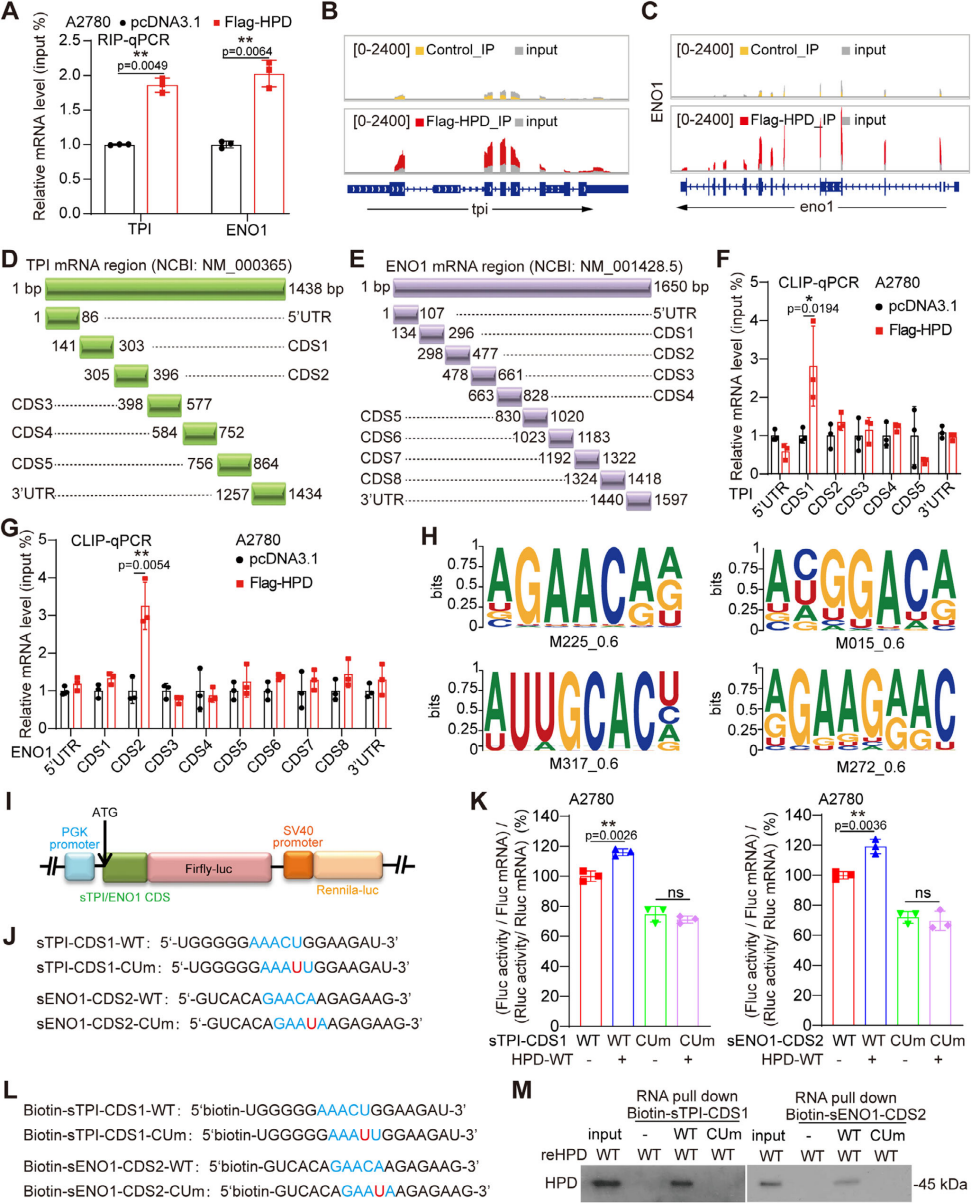
HPD directly binds to targeted mRNA through RRACH motif. A) RNA immunoprecipitation (RIP) assay was performed to detect the binding between HPD and mRNA of TPI or ENO1. B,C) Peaks showing HPD binding to mRNAs of TPI and ENO1 in A2780 cells, as measured by RIP‐seq. D,E) A schematic diagram of random segmentation of *TPI* and *ENO1* mRNA. F,G) CLIP‐qPCR was performed to explore the mRNA region of TPI and ENO1 bound by HPD. H) RNA binding motif of HPD was analyzed based on the RIP‐seq data. I) Schematic diagram of Firefly Luciferase. J) The sequence of short TPI‐CDS1 (sTPI‐CDS1) and short ENO1‐CDS2 (sENO1‐CDS2) for the construction of the reporter. K) Tethering assay was performed to measure translation efficiency of reporter mRNAs. Firefly luciferase (FLuc) activity was measured and normalized to the Renilla luciferase (RLuc) activity. Relative FLuc activity and RLuc activity were normalized to the relative FLuc and Rluc mRNAs, respectively. L) The sequence of biotin labelling sTPI‐CDS1 and sENO1‐CDS2. M) RNA pulldown was performed to detect the binding between HPD and biotin‐labeled mRNA of TPI or ENO1. Error bars in A, F, G, and K, mean values ± SD, *p* values were determined by unpaired two‐tailed Student's *t* test of *n* = 3 independent biological experiments. ^*^ *p* < 0.05; ^**^ *p* < 0.01; ^***^ *p* < 0.001.

To explore the mechanism of HPD as a non‐canonical RBP to promote translation, we first determined that HPD binds to mRNA of TPI and ENO1 by RNA immunoprecipitation (RIP) (**Figure** **3**A). According to RIP‐seq data, we found that HPD mainly binds to the CDS regions of TPI and ENO1 mRNA (Figure 3B,C). To locate the exact region which bound by HPD, we randomly divided the mRNA of TPI and ENO1 into several segments, each of which is ≈180 bp in length (Figure 3D,E). And then, we determined that HPD binds to the CDS1 region of TPI and the CDS2 region of ENO1 by crosslinking‐immunoprecipitation and qPCR (CLIP‐qPCR) (Figure 3F,G). Next, we analyzed the binding motif of HPD based on RIP‐seq data (Supplemental Table 4), and found that many of the binding motifs contained the RRACH (Purine‐Purine‐Adenine‐Cytosine‐Purine) motif, which is an m6A motif (Figure 3H). Therefore, we constructed the luciferase reporter with 18 bp sequences around the RRACH motif in the TPI‐CDS1 and ENO1‐CDS2 cloned upstream of firefly luciferase (Figure 3I). Then, we mutated RRACH to RRAUH to test whether HPD binds to mRNA through this motif (Figure 3J). The results showed that HPD increased the short TPI‐CDS1‐WT (sTPI‐CDS1‐WT) or short ENO1‐CDS2‐WT (sENO1‐CDS2‐WT) tagged reporter activity, but not the RRAUH mutant tagged reporter activity (Figure 3K). Lastly, to investigate whether HPD directly binds to mRNA, we synthesized biotin‐labeled wild type and mutant RNA of TPI‐CDS1 and ENO1‐CDS2 (Figure 3L). Through in vitro RNA pulldown, we found that only wild‐type RNA, but not the mutant RNA, was able to bind to the purified recombinant HPD (reHPD) (Figure 3M). The above results indicate that HPD directly binds to the targeted mRNA through the RRACH motif.

### 2.4 HPD Binds to mRNA Depending on its RBD Domains

**Figure 4:**
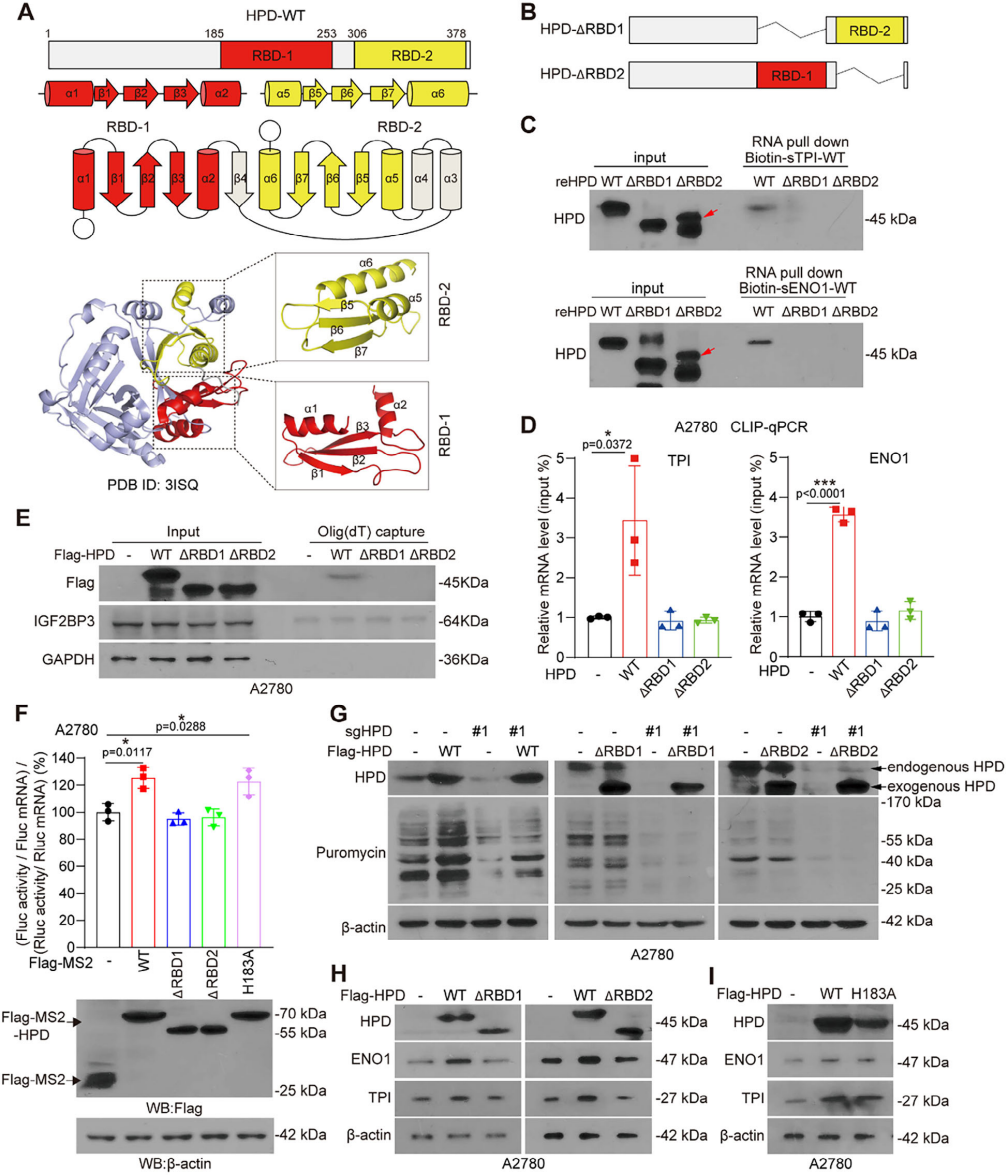
HPD binds to mRNA depending on its RBD domains. A) Ribbon representations of the structure of RBD (RNA binding domain) domain of HPD. B) Schematic diagram of the construction of truncated HPD. C) RNA pulldown was performed to detect the binding between HPD‐WT/truncated mutant and biotin‐labeled short mRNA of TPI/ENO1. The red arrow indicates reHPD‐△RBD2. D) CLIP‐qPCR was performed to detect the binding between HPD‐WT/truncated mutant and biotin‐labeled mRNA of TPI/ENO1. E) The levels of Flag, IGF2BP3, and GAPDH in eluates from A2780 cells captured by oligo (dT)_25_ were analyzed by western blotting. F) Tethering assay was performed to measure translation efficiency of reporter mRNAs. Firefly luciferase (FLuc) activity was measured and normalized to the Renilla luciferase (RLuc) activity. Relative FLuc activity and RLuc activity were normalized to the relative FLuc and Rluc mRNAs, respectively. G) The puromycylation assay was performed to detect global translation efficiency after rescuing Flag‐HPD WT or HPD RBD truncated mutants in HPD knockout A2780 cells. H,I) The levels of TPI and ENO1 were detected by western blotting in A2780 cells with exogenous expression of Flag‐HPD‐WT, Flag‐HPD‐△RBD1, Flag‐HPD‐△RBD2, and HPD‐H183A. Error bars in D and F, mean values ± SD, *p* values were determined by unpaired two‐tailed Student's *t* test of *n* = 3 independent biological experiments. ^*^ *p* < 0.05; ^**^ *p* < 0.01; ^***^ *p* < 0.001.

Next, we explore which domain of HPD is responsible for the association with mRNA. First, we analyzed the secondary and tertiary structure of HPD protein and found that there is a pair of double‐strand RNA binding domains (dsRBDs) containing α‐β‐β‐β‐α fold^[^ ^24^ ^]^ on HPD (**Figure** **4**A). Then, we used RNA Folding Form^[^ ^25^ ^]^ to predict the RNA structure of the RRACH motif on TPI‐CDS1 and ENO1‐CDS2, and confirmed that the RNA around this motif has a double‐stranded structure, which is consistent with the structure of mRNA bound by dsRBDs (Figure S3A, Supporting Information). Therefore, we speculated that the two dsRBDs (RBD1 and RBD2) on HPD play a role in binding RNA. To verify this hypothesis, we constructed HPD RBD1 or RBD2 motif deleted mutants, including HPD‐△RBD1 and HPD‐△RBD2 (Figure 4B). Then, we found that the ability of HPD to bind to mRNA was abolished when either RBD1 or RBD2 motif is deleted in in vitro (Figure 4C) and in vivo assay (Figure 4D,E; Figure S3B, Supporting Information), these results demonstrated that HPD directly binds to mRNA through its RBDs.

HPD recruits 4‐hydroxyphenylpyruvic acid (4‐HPPA) by H183 site as substrate to exert its metabolic tyrosine activity.^[^ ^26^ ^]^ Thus, we wonder whether the regulatory function of HPD on translation is dependent of its metabolic tyrosine activity. We first constructed 4‐HPPA binding site mutant vector and examined the activity of HPD as a catalytic enzyme in the tyrosine metabolic pathway. The results showed that RBD1/2 truncations and H183A mutant of HPD lost the activity that catalyzes the conversion of 4‐HPPA to HGA (Figure S3C, Supporting Information). Next, through tethering assays, we found that HPD could not promote global mRNA translation when the RBDs domains were deleted, and the inactivated mutant HPD‐H183A could still promote translation efficiency (Figure 4F). We further demonstrated that HPD did not promote mRNA translation after deletion of RBD1 or RBD2 by puromycylation assays (Figure 4G; Figure S3D, Supporting Information). In addition, HPD also lost the function of regulating the translation of TPI and ENO1 (Figure 4H; Figure S3E, Supporting Information), but the inactivated mutant HPD‐H183A still exerts the regulation effect on the protein levels of TPI and ENO1 (Figure 4I). These results suggested that the function of HPD in promoting translation is independent of its metabolic enzyme activity.

To further confirm the role of HPD in promoting translation, we tested whether HPD might interact with translational factors. To this end, we performed co‐immunoprecipitation (co‐IP) experiments with FLAG‐affinity‐purified complexes and tested for the association of HPD with proteins by mass spectrometry. The data showed that some ribosomal proteins, such as RPS10, RPL10, RPL20, may interact with FLAG‐HPD. (Figure S3F, Table S5, Supporting Information). Then, we selected RPS10 obtained by mass spectrometry for verification, and two translation factors EIF3A and EIF3B were used as negative controls. We found that RPS10 indeed binds to Flag‐HPD (Figure S3G, Supporting Information), while EIF3A and EIF3B could not (Figure S3H, Supporting Information). To a certain extent, this shows that HPD binds specifically to some translation‐related factors. In summary, these results suggested that HPD relies on its RBD domains to bind mRNA, as well as recruits ribosomal proteins to promote translation efficiency, and this function is independent of its metabolic tyrosine activity.

### 2.5 HPD Promotes Ovarian Cancer Glycolysis and Tumor Growth in a RBP‐Dependent Manner

**Figure 5:**
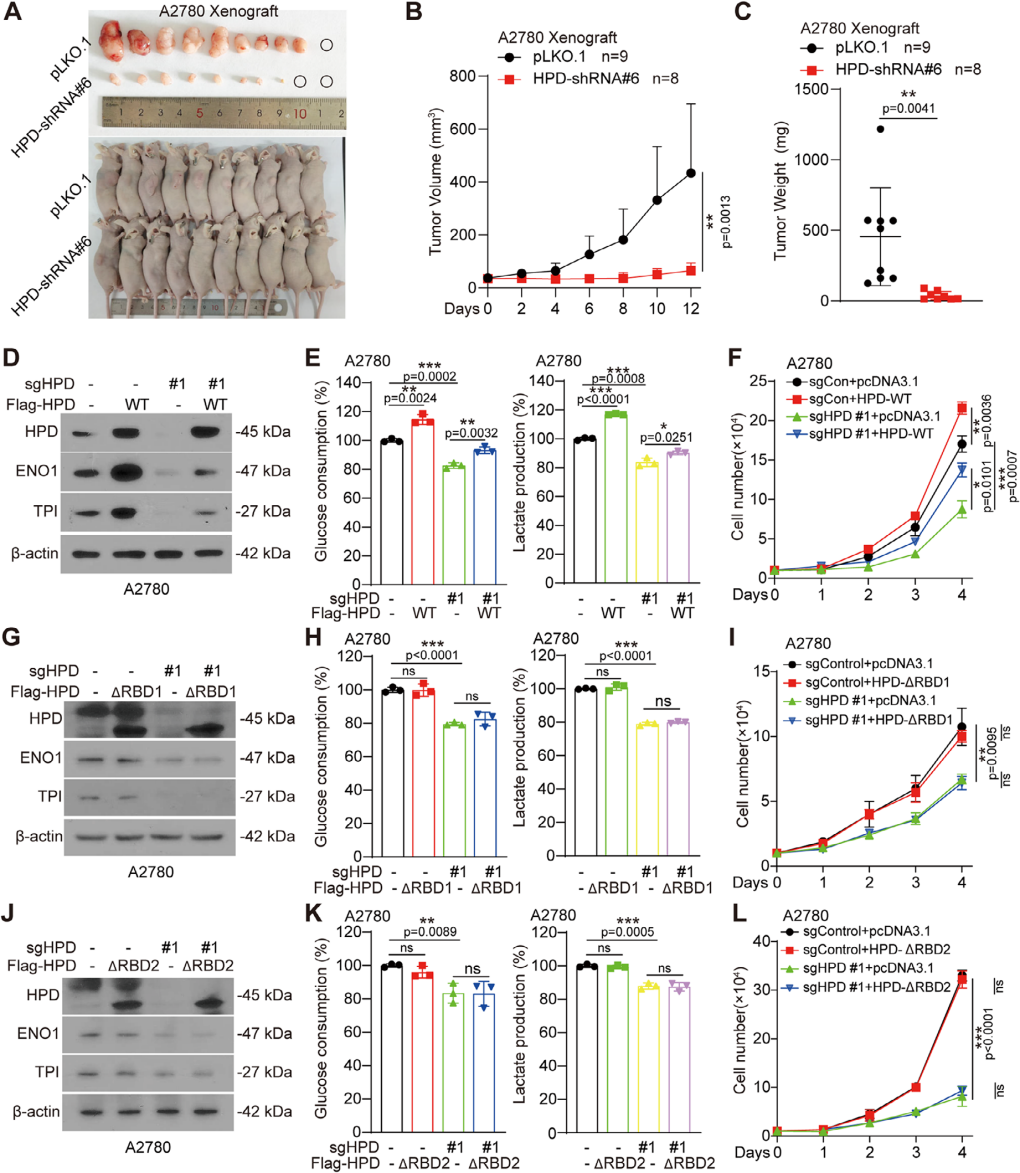
HPD promotes ovarian cancer glycolysis and tumor growth in a RBP‐dependent manner. A) The tumors in xenograft nude mouse are shown. B) The tumor growth curve was recorded in xenograft nude mice bearing A2780 cells tumor with HPD knockdown. C) Tumor mass was examined in xenograft nude mice bearing A2780 cells tumor with HPD knockdown. D) The expression level of HPD was detected in HPD knockout A2780 cells, which were exogenously expressed with or without Flag‐HPD‐WT. E) The relative glucose consumption and lactate level were determined in A2780 cells with HPD knockout, which were exogenous express with or without Flag‐HPD‐WT. F) The cell proliferation was determined by cell number counting in A2780 cells with HPD knockout, which exogenous express with or without Flag‐HPD‐WT. G) The expression level of HPD was detected in HPD knockout A2780 cells, which exogenously expressed with or without Flag‐HPD‐△RBD1. H) The relative glucose consumption and lactate level were determined in A2780 cells with HPD knockout, which exogenous express with or without Flag‐HPD‐△RBD1. I) The cell proliferation was determined by cell number counting in A2780 cells with HPD knockout, which exogenous express with or without Flag‐HPD‐△RBD1. J) The expression levels of HPD were detected in HPD knockout A2780 cells, which exogenous express with or without Flag‐HPD‐△RBD2. K) The relative glucose consumption and lactate level were determined in A2780 cells with HPD knockout, which exogenous express with or without Flag‐HPD‐△RBD2. L) The cell proliferation was determined by cell number counting in A2780 cells with HPD knockout, which exogenous express with or without Flag‐HPD‐△RBD2. Error bars in E, F, H, I, K, and L, mean values ± SD, p values were determined by unpaired two‐tailed Student's *t* test of *n* = 3 independent biological experiments. ^*^ *p* < 0.05; ^**^ *p* < 0.01; ^***^ *p* < 0.001.

Given that HPD controls translation regulation of ENO1 and TPI, we next wanted to find out whether ENO1 and TPI are involved in glycolysis and cell proliferation in HPD‐mediated RBP‐dependent manner in human ovarian cancer. First, we examined the effect of HPD on glucose consumption, lactate production, and cell proliferation, and found that knockout or knockdown of HPD inhibited glucose consumption, lactate production, and cell proliferation (Figure S4, Supporting Information). In addition, we found that HPD knockdown significantly inhibited tumor growth in vivo (**Figure** **5**A–C; Figure S5A–C, Supporting Information). The protein levels of TPI and ENO1 were significantly decreased in the HPD knockdown tumor tissues (Figure S5D,E, Supporting Information).

Then, exogenous expression of ENO1 or TPI in HPD knocked down cells partially restored glucose consumption and lactate production, as well as cell proliferation (Figure S6, Supporting Information). This indicates that HPD affects the glycolysis pathway and cell proliferation by regulating ENO1 and TPI. These results suggested that HPD promotes glycolysis and cell proliferation by regulating TPI and ENO1.

To investigate whether the effect of HPD on glycolysis and cell proliferation is dependent on its RBP function, we exogenous expressed HPD‐WT, HPD‐△RBD1, and HPD‐△RBD2 in HPD knocked out cells and found that HPD‐WT partially rescued the decrease of ENO1 and TPI protein levels (Figure 5D; Figure S7A, Supporting Information), as well as glucose absorption, lactate production, and cell proliferation (Figure 5E,F; Figure S7B,C, Supporting Information). However, HPD without RBDs lost this ability (Figure 5G–L; Figure S7D–I, Supporting Information). These results suggest that HPD promotes glycolysis and cell proliferation in a RBP‐dependent manner.

### 2.6 Targeting HPD RNA Biding Ability Abrogates Tumor Growth and Enhances Taxol Response

**Figure 6:**
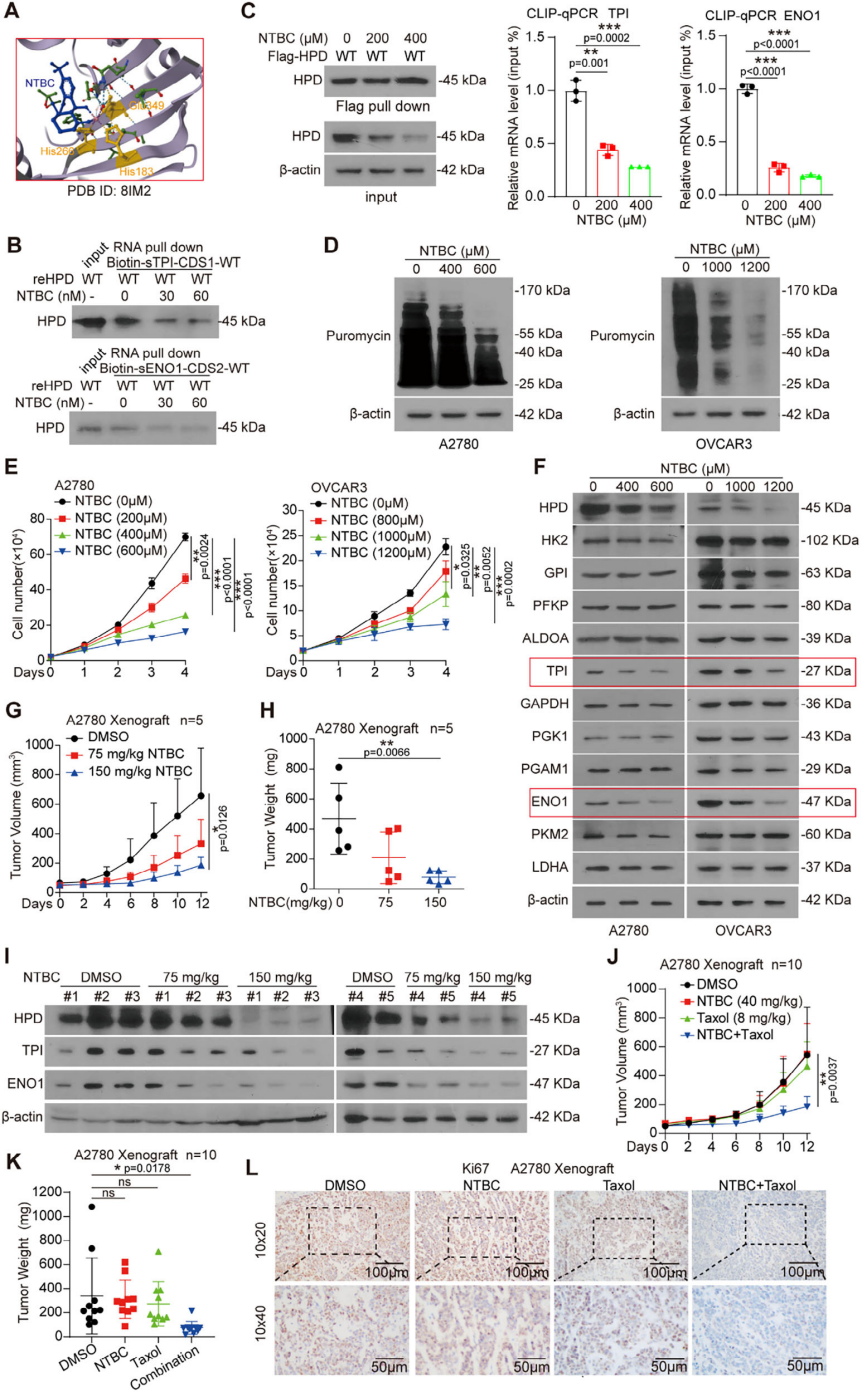
Targeting HPD RNA binding ability abrogates tumor growth and enhances Taxol response. A) The crystal structure diagram of the combination of NTBC and HPD. B) The RNA pulldown was performed to detect the binding of reHPD‐WT and mRNA of TPI/ENO1 treated with or without NTBC in vitro. C) CLIP‐qPCR was performed to detect the binding of Flag‐HPD‐WT and mRNA of TPI/ENO1 treated with or without NTBC in A2780 cells. D) The puromycylation assay was performed to detect global translation efficiency in ovarian cancer cells treated with NTBC. E) The cell proliferation was determined by cell number counting in ovarian cancer cells treated with different concentrations of NTBC. F) The levels of glycolytic enzymes were detected by western blotting in ovarian cancer cells treated with different concentrations of NTBC. G) Tumor growth curves were recorded in xenograft nude mice bearing A2780 cells tumor treated with NTBC. H) Tumor mass was examined in xenograft nude mice bearing A2780 cells tumor treated with NTBC. I) The levels of HPD, ENO1, and TPI were detected by western blotting in the tumor treated with NTBC. J) Tumor growth curve was recorded in xenograft nude mice bearing A2780 cells tumor treated with NTBC or Taxol. K) Tumor mass was examined in xenograft nude mice bearing A2780 cells tumor treated with NTBC or Taxol. L) The IHC was performed to detect the level of Ki67 in the representative tumor. Error bars in C and E, mean values ± SD, p values were determined by unpaired two‐tailed Student's *t* test of *n* = 3 independent biological experiments. ^*^ *p* < 0.05; ^**^ *p* < 0.01; ^***^ *p* < 0.001.

Many literatures reported that 2‐(2‐nitro‐4‐trifluoromethylbenzoyl)‐1, 3‐cyclohexanedione (NTBC) is an effective inhibitor of HPD, and we were excited to discover that many of the binding sites of NTBC on HPD are located on two RBDs, such as His266, Glu349, and so on^[^ ^27^ ^]^ (**Figure** **6**A). Thus, we wonder whether NTBC disrupts the interaction between HPD and mRNA, then, we performed in vitro RNA pulldown and observed that the NTBC disrupted the binding of HPD to mRNA (Figure 6B). Next, we also obtained consistent results in A2780 cells (Figure 6C). Meanwhile, the NTBC does not induce degradation of the bait RNA or diminishes the levels of input HPD (Figure S8A, Supporting Information). Furthermore, we found that treatment of NTBC inhibited the global translation (Figure 6D). In addition, NTBC inhibited the proliferation of ovarian cancer cells (Figure 6E) and down‐regulated the protein levels of TPI and ENO1 in ovarian cancer cells (Figure 6F). Finally, NTBC inhibited the development of ovarian cancer in A2780 CDX mouse model (Figure 6G,H; Figure S8B,C, Supporting Information) and reduced the protein levels of TPI and ENO1 (Figure 6I; Figure S8D, Supporting Information).

Taxol is a natural anti‐cancer drug, which has been widely used in the clinical treatment of ovarian cancer. However, there are several different mechanisms to induce resistance of the cancer cells to Taxol. In this study, we explored whether targeting HPD's RBP‐dependent function could increase the sensitivity of ovarian cancer to Taxol treatment. Indeed, in an A2780 CDX mouse model, we found that the combination of NTBC and Taxol had a better inhibitory effect on the growth of ovarian cancer (Figure 6J–L; Figure S8E,F, Supporting Information). Furthermore, we generated patient‐derived ovarian cancer organoid models and found that NTBC effectively suppressed their growth (S8G). Based on the above results, we determined that targeting HPD with small molecule inhibitors blocks its binding to mRNA and inhibits the development of ovarian cancer, as well as enhances the Taxol response, which provides a new theoretical basis for clinical medication.

### 2.7 HPD is Significantly Correlated with the Protein Levels of TPI and ENO1 in Ovarian Cancer

**Figure 7:**
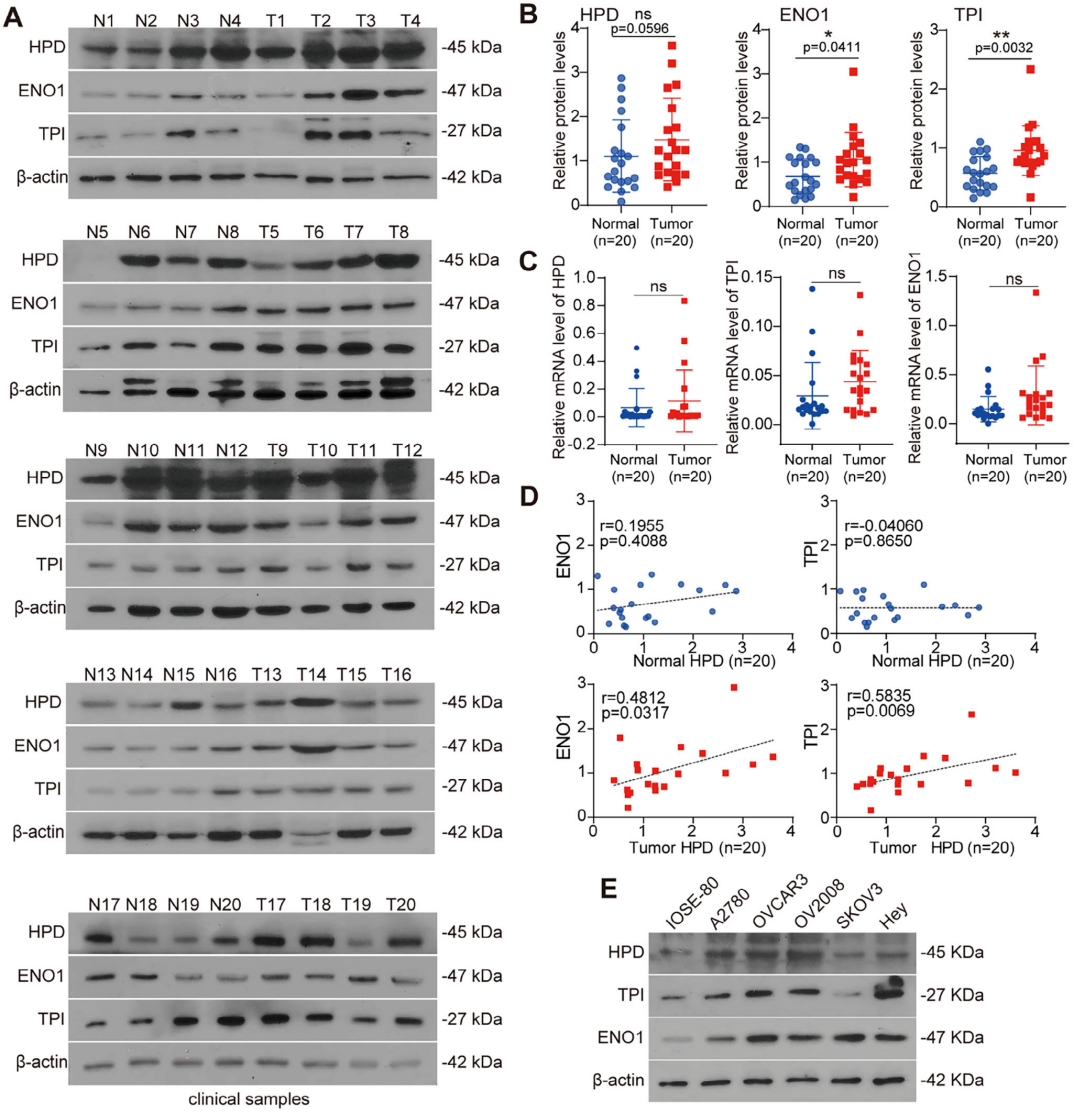
HPD is significantly correlated with the protein levels of TPI and ENO1 in ovarian cancer. A) The levels of HPD, ENO1, and TPI were detected by western blotting in clinical normal ovarian tissues and ovarian cancer tissues. B) The levels of HPD, ENO1, and TPI were analyzed in clinically normal ovarian tissues and ovarian cancer tissues. C) The mRNA levels of HPD, ENO1, and TPI were detected by qPCR assay in clinical normal ovarian tissues and ovarian cancer tissues. D) The correlation between HPD and ENO1/TPI protein expression in clinical samples was analyzed. E) The levels of HPD, ENO1, and TPI were detected by western blotting in various human ovarian cancer cells (A2780, OVCAR3, OV2008, SKOV3, and Hey) and normal ovarian cells (IOSE‐80). Error bars in B and C, mean values ± SD, *p* values were determined by unpaired two‐tailed Student's *t* test. ^*^ *p* < 0.05; ^**^ *p* < 0.01; ^***^ *p* < 0.001.

Given that HPD controls translation regulation of TPI and ENO1, we next wanted to find out whether TPI and ENO1 are co‐expressed with HPD in human ovarian cancer. We collected 20 normal ovarian tissues and 20 ovarian cancer tissues, and detected the protein levels of HPD, TPI, and ENO1 by WB (**Figure** **7**A). We observed a slight but not significant increase in HPD in tumor tissues compared to normal tissues, and the expression of ENO1 and TPI was significantly higher in tumor tissues than in normal ovarian tissues (Figure 7B). The mRNAs levels of HPD, TPI, and ENO1 were not significantly different between tumor tissues and normal ovarian tissues (Figure 7C). This hints that the increase of TPI and ENO1 at the protein level is caused by the improvement of translation efficiency mediated by HPD. More importantly, we found that the protein levels of TPI and ENO1 were positively correlated with HPD in ovarian cancer tissues, but not in normal ovarian tissues (Figure 7D). Furthermore, as the protein level of HPD increases in ovarian cancer cell lines, so does the protein level of TPI and ENO1 (Figure 7E). In summary, HPD is positively correlated with the protein levels of TPI and ENO1, which play an important role in the progression of ovarian cancer.

## 3 Discussion

HPD is known as a key enzyme in the tyrosine catabolism pathway, which is involved in metabolizing 4‐HPPA to HGA, and its deficiency usually leads to the hereditary type III tyrosinemia.^[^ ^28^ ^]^ Emerging evidence has revealed that the dysregulation of HPD expression also as a driver in breast cancer, lung cancer, and liver cancer progression.^[^ ^8^, ^29^, ^30^ ^]^ Although the non‐metabolic functions of HPD in cancer progression have been explored, it remains poorly understood that more non‐metabolic functions of HPD are related to cancer and disease. It is therefore intriguing to discover the importance moonlighting function of HPD as a RBP in the regulation of mRNA translation, particularly several mRNAs involved in ovarian cancer glycolysis.^[^ ^31^ ^]^ Cancer cells preferably rely on aerobic glycolysis rather than oxidative phosphorylation. In this respect, translational control of glycolysis enzymes mRNA by tyrosine catabolism enzyme HPD, which may be a fast way of controlling cancer progression by reprogram aerobic glycolysis. The non‐canonical RBP funciton of HPD may provide extra advantages for the fitness of ovarian cancer cells. Thus, our findings amplify the effects of cancer drivers in promoting tumor progression, including ovarian cancer.

Employing RIP, we uncovered an oncogenic program comprised of thousands of mRNAs bound by HPD through RRACH motif, and applied Cross‐linked RNA Immunoprecipitation (CLIP) confirming that the binding motif of HPD to mRNA was RRACH on CDS region. In addition, as reports show that RBPs bind the CDS region may affect the secondary structure of mRNA and thus promotes mRNA translation.^[^ ^32^ ^]^ In order to explore the effect of HPD on its binding mRNAs, we are intrigued to discover that HPD promotes mRNA translation, in particular glycolytic enzymes TPI and ENO1. Furthermore, employing mass spectrometry analysis, we identify several ribosomal complex proteins that are bound by HPD, which coordinate HPD to promote mRNA translation. Lastly, we found that the binding of HPD to RNA depends on its two dsRBDs. These two dsRBDs form a pocket, so they are indispensable for HPD to bind to RNA. Interestingly, this pocket is also the active center of HPD as a metabolic enzyme. This phenomenon exists in the case of many metabolic enzymes performing non‐metabolic functions. For example, Huppertz et al. reported that ENO1 has RNA‐mediated inhibition of enzyme activity in stem cells.^[^ ^33^ ^]^ While ENO1 functions as an RNA‐binding protein, its RNA binding function relies on its DNA‐binding domain.^[^ ^17^ ^]^ This study demonstrates that HPD's RNA binding function is dependent on its double‐stranded RNA‐binding domain. Notably, both HPD and ENO1 share the common feature of enhancing mRNA translation levels after binding to mRNA. In addition, in order to try to reveal the metabolic function of HPD and the function of binding RNA, we mutated the substrate‐binding site H183 of HPD, which is located outside the two dsRBDs, and still observed that HPD promoted TPI and ENO1. To a certain extent, this indicates that the function of HPD to exert RBP is independent of enzyme activity.

The emergence of recent technological advancements, such as genomics and RNA sequencing (RNA‐seq) to the exploration and identification of hitherto unknown biomarkers.^[^ ^34^ ^]^ However, the progress of translation from lower abundance mRNA to higher abundance protein limits the exploration and identification of lower abundance mRNA as prognostic biomarkers. Thus, it is urgent to explore the role and mechanism of the driver in regulating mRNA translation. The mRNA translation function of RBPs, which provides a good choice to develop small RNA molecules based on the recognition motif on mRNA by RBPs to treat disease, including cancer. Historically, glycolysis is a main energy provider in ovarian cancer growth, invasion, and migration.^[^ ^35^ ^]^ Thus, lots of agents targeting the glycolysis enzymes have been developed to attenuate tumor progression and overcome drug resistance.^[^ ^36^, ^37^ ^]^ However, they do not play a significant role in ovarian cancer. Considering that the RNA binding pocket of HPD is in the same position as the active center of the catalytic enzyme, we tried to use the enzyme activity inhibitor NTBC^[^ ^38^ ^]^ of HPD to inhibit its RBP function. Specifically, molecular docking simulations demonstrated that NTBC occupies the RNA‐binding pocket of HPD, sterically hindering potential interactions with target mRNA. To further validate this finding, we performed both in vitro and in vivo experiments, including RNA pull‐down and CLIP‐qPCR, which confirmed that NTBC effectively disrupts the association between HPD and its target mRNA. These results collectively support our conclusion that NTBC acts as a competitive inhibitor by blocking the RNA‐binding interface of HPD. At the same time, NTBC showed inhibitory effects on ovarian cancer in vitro and in vivo, as well as enhanced the Taxol response. These results suggested that it is a good choice to develop small RNA molecules based on the recognition motif of HPD for RNA interference (RNAi) of the RBP function of HPD. However, although we have demonstrated that NTBC block the binding of HPD to target mRNA in vivo and in vitro, NTBC, as a drug targeting the metabolic function of HPD, cannot accurately indicate whether its inhibitory effect on ovarian cancer is achieved by blocking the RBD function of HPD or by inhibiting the activity of metabolic enzymes. From a clinical point of view, it will be safer to develop inhibitors that specifically block the RBP function of HPD without affecting its metabolic enzyme activity. Specific small RNA molecules were developed according to the motif recognized by HPD may be a potential drug development candidate for ovarian cancer treatment.

## 4 Conclusion

We identified the moonlighting function of HPD, which exerts as a non‐canonical RBP to promote translation in ovarian cancer. Mechanically, HPD binds to the RRACH motif in the CDS region of mRNA, depending on its own two dsRBDs. Furthermore, HPD increases the translation level of glycolytic enzymes, TPI and ENO1, to promote the progression of ovarian cancer. This work provides new evidence for HPD as RBP, which binds to the CDS region of mRNA, and gives a new theoretical basis and scientific significance for exploring the non‐classical functions of metabolic enzymes. Meanwhile, we also provide a new target for the treatment of ovarian cancer.

Although our combined data suggest that HPD as an RBP increases global mRNA translation by binding to the RRACH motif of mRNA through its own two dsRNA binding domains, further mechanism is required to reveal the molecular mechanism of HPD promoting translation. Of note, our IP‐MS analysis showed that many proteins interacting with HPD are related to the regulation of translation initiation, translation activator activity, cytoplasmic translation, and so on (Figure S3F; Table S5, Supporting Information). It is also unclear whether HPD directly binds to these proteins to be involved in the translation process. Another hypothesis is whether HPD directly binds to mRNA affects the translation elongation by changing the structure of mRNA. Further studies are needed to confirm these results and reveal the underlying mechanisms.

## 5 Experimental Section

### Cell Lines

A2780 cells were purchased from JiNiOu (Guangdong, China). IOSE‐80, Hey, OVCAR3, SKOV3, and HEK293T cells were purchased from Procella (Wu Han, China). HEK293T and OVCAR3 cells were maintained in Dulbecco's modified Eagle's medium (DMEM, Thermo Fisher Scientific, MA, USA) supplemented with 10% fetal bovine serum (FBS, ExCell Bio, Shanghai, China). IOSE‐80, A2780, Hey, and SKOV3 cells were cultured in RPMI‐1640 medium (Thermo Fisher Scientific, MA, USA) supplemented with 10% FBS. Human ovarian surface epithelial cancer cell, OV2008, was a gift from Dr. Benjamin K. Tsang (University of Ottawa, Ontario, Canada) and was cultured in RPMI‐1640 medium with 10% FBS. All cells were cultured at 37 °C in an atmosphere of 5% CO_2_/95% air (normoxic conditions) at 37 °C.

### Animal Model

To establish A2780 cell‐derived xenografts, 5 × 106 A2780 cells were inoculated subcutaneously into the right flank of female BALB/c nude mice (4–6 weeks old, purchased from Charles River, Beijing, China). Tumor growth was monitored every 2–3 days using calipers. When the tumor sizes reached ≈4 × 4 mm, the mice were randomly divided into experimental groups. The viral suspension of pLKO.1 (control vector) or shHPD was injected directly into the tumor at a volume of 25 µL per injection, once a day for a total of 5 consecutive days. For the compound treatment assay, the compound solutions (dissolved in DMSO) were administered to the nude mice via intraperitoneal injection at a dose indicated in the figures, once every two days. The control group received an equivalent volume of vehicle (DMSO) following the same schedule. The animal studies were performed according to the institutional ethical guidelines.

### Western Blot

For Western blot, treated cells were collected and fully lysed in NP‐40 lysis buffer. The lysates were separated on sodium dodecyl sulfate 8–12% polyacrylamide gel electrophoresis and transferred onto a nitrocellulose membrane, and specific proteins were detected by enhanced chemiluminescence. All the antibodies used in this paper are listed in KEY RESOURCES TABLE.

### Cell Proliferation Assay

First, the cells were inoculated into 24‐well plates, and 1 × 10^4^ cells were inoculated into each well. For the cells that need to be treated with drugs, after 24 h of cell inoculation, the corresponding drugs were added to the wells to treat the cells. Then, cells were collected every 24 h after dosing, and cell counts were performed by manual counting under light microscopy using a hemocytometer. After four days, the cell survival rate was calculated, and the cell growth curve was drawn.

### RNA Pulldown Assay

For RNA pulldown assay, biotin‐labeled RNA chains were synthesized from GENEWIZ (Suzhou, China). An RNA pulldown assay was performed with Streptayidin Agarose Resin (Thermo Fisher). The Streptayidin Agarose Resin was first washed with pre‐cooled PBS. Then, 10 µm RNA, 1 µg protein samples, and 50 µL Streptayidin Agarose Resin were mixed in 1 mL PBS and rotated them overnight at 4 °C. The mixed Streptayidin Agarose Resin was washed with PBS three times and detected by western blot.

### mRNA Stability Assay

Actinomycin D was a cell cycle non‐specific drug, which can inhibit DNA repair and RNA transcription, and transport. The principle of Actinomycin D was to form a stable complex between guanine and cytosine (G‐C) base pairs embedded in the double helix of DNA, thereby hindering the function of RNA polymerase and preventing the synthesis of RNA, especially mRNA. To perform mRNA stability, Actinomycin D (5 µg mL^−1^) was treated to cells for the indicated times, and the mRNA levels at each time point were analyzed by qPCR.

### Protein Stability Assay

Cycloheximide (CHX) was used as a protein synthesis inhibitor in eukaryotes. The main principle of CHX was to block the elongation stage of eukaryotic translation by binding ribosomes and inhibiting eEF2‐mediated translocation. After CHX binds to ribosomes, it prevents ribosomes from moving on mRNA, thereby preventing the continuation of protein synthesis. To detect the protein stability, CHX was used to treat cells at 300 µm final concentration for the indicated times. The cells were collected and lysed with lysis buffer. The protein levels were detected by Western blot.

### Gene Knockdown with shRNA and Gene Knockout with sgRNA

Lentiviral vectors harboring shRNA were purchased from Transheep Biological Corporation (Transheep, Shanghai, China) or lentiCRISPR v2 with sgRNA from Tsingke Biotechnology Co (Beijing, China) for stable knockdown or knockout of endogenous HPD in cells. The packing plasmids, including pMD2.G and psPAX2, were co‐transfected with lentiviral vectors into HEK293T cells. After filtering through 0.45‐µm filter, the lentiviral particles were infected into A2780 or OVCAR3 cells, and stable knockdown or knockout cell lines were selected with puromycin.

### Plasmid Constructs and Transfections

Human HPD cDNA (NM_001171993.2) and TPI cDNA (NM_000365.6) were cloned into pcDNA3.1 with Flag‐tag at N‐terminus or pETM3C vector with His‐tag. The HPD‐△RBD1 or ‐△RBD2 were constructed using the Hieff Clone® Plus One Step Cloning Kit (YEASEN, Shanghai, China). ENO1 cDNA (NM_001428.5) was cloned into pLVX3 with 3X Flag‐tag ADDIN EN.CITE (Xie et al., 2023). FLuc‐MS2bs, pFlag‐MS2, and pFlag‐MS2‐GW182 C‐term plasmids were gifts from professor Shuibin Lin (Center for Translational Medicine, The First Affiliated Hospital, Sun Yat‐sen University, Guangzhou, China). The HPD fragment was subcloned into the NotI and BamHI sites of pFlag‐MS2. For transfections, polyethylenimine (PEI) was use to transfect plasmids into cells after growing to 80% density. All cloning primers are listed in KEY RESOURCES TABLE.

### Glucose Consumption and Lactate Production Assays

For glucose consumption and lactate production assays, the treated cells were washed with PBS after discarding the medium and cultured in phenol red‐free medium for 1 h. Glucose and lactate in the recovered medium were detected by the Glucose kit (A154, Nanjing Jiancheng Bioengineering Institute, Nanjing, China) and the Lactic Acid assay kit (A019, Nanjing Jiancheng Bioengineering Institute, Nanjing, China), respectively. The remaining cells were collected and counted to normalize the relative glucose consumption and lactate production.

### RNA Extraction and RT‐qPCR

TRIzol (15596018, Gibco) was used to isolate total RNA of cells or tissue samples. Hifari III 1st Strand cDNA Synthesis SuperMix for qPCR (11141ES50, YEASEN, Shanghai, China) was used to synthesize cDNA, and real‐time quantitative PCR (RT‐qPCR) was performed by Hieff qPCR SYBR Green Master Mix.

### Identification of mRNA‐Interacting Proteins

In order to isolate the proteins interacting with mRNA, four 10 × 10 cm^2^ cells were washed twice by iced PBS and irradiated with 0.15 J cm^−2^ UV light at 254 nm.^[^ ^10^, ^19^, ^20^ ^]^ Cells were harvested and lysed in lysis/binding buffer (100 mM Tris‐HCl, pH 7.5, 500 mM LiCl, 0.5% Lithium Dodecyl Sulfate (LiDS), 1 mm EDTA, 5 mm DTT). After homogenizing with a 0.4‐mm diameter needle, the lysates were gently rotated with 1 mL of oligo (dT)_25_ magnetic beads (S1419S, New England Biolabs) at 4 °C overnight. According to the instructions, the magnetic beads were washed with wash buffer I (20 mm Tris‐HCl, pH 7.5, 500 mm LiCl, 0.1% LiDS, 1 mm EDTA, 5 mm DTT), wash buffer II (20 mm Tris‐HCl, pH 7.5, 500 mm LiCl, 1 mm EDTA) and low‐salt buffer (20 mm Tris‐HCl, pH 7.5, 200 mm LiCl, 1 mm EDTA) in turn. mRNA‐protein complexes were eluted from beads in elution buffer (20 mm Tris‐HCl, pH 7.5, 1 mm EDTA). For RNA analysis, RNA was extracted by TRIzol. For protein analysis, samples were added SDS‐loading buffer and boiled, followed by western blot.

### RNA Immunoprecipitation (RIP) and Cross‐linked RNA Immunoprecipitation (CLIP)

To obtain the HPD‐bound RNA, four 10 × 10 cm^2^ cells overexpressing Flag‐HPD were washed twice with iced PBS. For CLIP, the cells were irradiated with 0.15 J/cm2 UV light at 254 nm and lysed with lysis buffer. If necessary, 1 U/ml RNase T1 (EN0601, Thermo Fisher) was used to digest lysates for 15 min at 37 °C. Then, the lysates were incubated with ANTI‐FLAG M2 affinity gel (A2220, Sigma) overnight at 4 °C. The RNA‐protein complexes were washed with TBS (50 mmol/L Tris, 150 mmol/L NaCl) and subjected to RNA extraction.

### RNA‐Immunoprecipitation Sequencing (RIP‐seq)

The RIP‐seq was performed by Wuhan SeqHealth Tech Co., Lid (Wuhan, China). The sequencing process was performed according to the company's instructions. In brief, the cells were lysed under mild conditions, and the Flag‐HPD was enriched by the Flag antibody, and then the Flag‐HPD‐binding RNA was recovered. RNA was extracted using TRIzol (Thermo Fisher, 15596018CN), quantified (Qubit 3.0), and integrity verified by electrophoresis. Libraries were prepared with UMI‐labeled KCTM Digital Stranded mRNA Library Prep Kit (Seqhealth Tech), and 200–500 bp fragments were sequenced on DNBSEQ‐T7 (PE150).

Raw data were processed by fastp (v0.23.1) to remove adapters/low‐quality reads. UMI‐based deduplication was performed: reads with identical UMIs were clustered, aligned (>95% identity), and collapsed into consensus sequences using custom scripts. Clean reads were aligned to the human genome (Ensembl release 87) via STAR (v2.7.6a). Peaks were called using exomePeak (v2.16.0), annotated with RnaPeakAnnotate (v1.0.9), and analyzed for distribution (deepTools v3.5.1). Differential peaks were identified by Fisher's test (Python script), and motifs were analyzed via HOMER (v4.9.1). Functional enrichment (GO/KEGG) was assessed using KOBAS (v2.1.1, P < 0.05).

### Protein Mass Spectrometry Analysis

In order to identify the proteins bound to HPD, the Novogene company (Beijing, China) was commissioned to carry out protein mass spectrometry analysis. First, Flag‐HPD was overexpressed in A2780 cells. After 48 h, the cells were collected and lysed. Flag antibody was used to immunoprecipitate (IP) the cell lysate, and IgG was used as the negative control to immunoprecipitate (IP) the same amount of cell lysate. The samples after IP were separated by polyacrylamide gel electrophoresis (SDS‐PAGE). The target protein strips were collected and sent to the company for qualitative analysis of protein mass spectrometry.

### Polysome Fractionation Assay

Polysome fractionation by sucrose density gradient centrifugation was performed to explore the translation mechanism regulated by HPD. Before collecting cell precipitates, two 10 × 10 cm2 cells were treated with 100 µg mL^−1^ cycloheximide (CHX) at 37 °C for 15 min and washed with PBS. After lysing and centrifuging the cell precipitate, the supernatant was collected and added 10 U mL^−1^ RNase inhibitor was added. The linear sucrose gradient solution of 10%–50% was prepared by the Biocomp density gradient preparation instrument. The supernatant was added to the sucrose density gradient solution. Ribosomal subunits and polyribosomes were separated in a gradient solution by ultracentrifugation at 4 °C for 2 h with a centrifugal force of 260 000. The distribution of ribosomes was analyzed by measuring the absorbance of each component at 260 nm optical density by Nanotrop. RNA from each fraction was extracted with TRIzol and detected by RT‐qPCR.

### Ribosome Profiling (Ribo‐seq)

The Ribo‐seq was performed by Wuhan SeqHealth Tech Co., Lid (Wuhan, China). The sequencing process was performed according to the company's instructions. In brief, the cells were seeded into 15 cm dishes before collection of cells. The cells were treated with 100 µg/mL cycloheximide (CHX) and incubated for 5 min at 37 °C and washed with PBS containing 100 µg mL^−1^ CHX. Subsequently, the cells were collected by cell scraping and centrifuged to obtain a precipitate. Total RNA was extracted for quality control (Nanodrop A260/A280; LabChip GX Touch integrity) and quantified (Qubit3.0, Q10210). Lysates were digested with RNase I, and ribosomes were enriched via MicroSpin S‐400 HR columns (Cytiva, GE27‐5140‐01). Ribosome‐protected fragments (RPFs, 26–32 nt) were isolated by PAGE (Zymo Small‐RNA™ Recovery Kit, R1070), followed by rRNA depletion (in‐house kit). Libraries were prepared using QIAseq miRNA Kit (Qiagen, 331502) and sequenced (Novaseq X Plus, PE150).

Raw reads were processed with fastp (v0.23.2) to remove adapters/low‐quality sequences, followed by rRNA/tRNA filtering (SortMeRNA v4.3.3). UMI‐based deduplication was performed: reads were clustered by UMI, sub‐clustered by >95% sequence identity, and collapsed into consensus sequences (custom scripts). Valid RPFs (20–40 nt) were aligned to the human genome (STAR v2.7.6a). Trinucleotide periodicity and codon usage were analyzed (riboWaltz v2.0), while translation pauses were predicted (PausePred v1.0.0). Gene‐level counts (featureCounts, Subread‐1.5.1) were normalized to RPKM for differential analysis (edgeR v3.40.2). Functional enrichment (GO/KEGG) was assessed (KOBAS v2.1.1, P<0.05).

### Tethering Assays

For tethering assays, a MS2‐fusion protein is co‐expressed with a luciferase reporter construct containing high‐affinity MS2‐binding sites (MS2bs) in its 3'‐UTR. GW182 C‐term is the control that suppresses translation in Tethering assays.^[^ ^39^, ^40^ ^]^ The firefly luciferase reporter with MS2bs (Fluc‐MS2bs), the plasmids expressing MS2‐fusion protein (MS2‐HPD or MS2‐GW182 C‐term) and control renilla luciferase reporter were co‐transfected into A2780 cells. 48 h after transfection, the cells were collected, one part was lysed with lysis buffer from Dual Luciferase Reporter Gene Assay Kit II (RG029, Beyotime), and the other part was extracted RNA to detect the mRNA level of the fluorescent reporter gene by qPCR. Firefly and Renilla luciferase activity were measured with this kit according to the manuscript instructions. Relative FLuc activity and RLuc activity were normalized to the relative FLuc and Rluc mRNAs, respectively.

### Puromycylation Assays

Puromycylation assay was used to analyze protein synthesis. The cells were seeded and incubated for 24 h. Then we treated the cells with the indicated drugs for 48 h. After that, the cells were cultured in medium containing 10 µg mL^−1^ puromycin at 37 °C for 15 min. The whole cell proteins were extracted, and WB assay was used to detect the puromycin incorporation into the nascent chain using a specific anti‐puromycin antibody.

### HPD Activity Assay

To detect the activity of HPD, the cloning plasmid of HPD was transformed into E.coli BL21 and cultured overnight. Monoclonal was picked to 5 mL of liquid LB medium and incubated overnight at 37 °C, 220 rpm. On the second day, 10 µL of bacterial liquid was transferred to a new 5 mL liquid LB medium with tyrosine, and incubated at 37 °C, 220 rpm for 3.5 h. IPTG with a final concentration of 1 mg mL^−1^ was added to the medium to induce protein expression. After 8 h of culture at 16 °C with shaking at 160 rpm, it was continued to be incubated overnight at 37 °C, 220 rpm. Finally, the supernatant of the bacterial liquid was collected, and the absorbance value was measured at a wavelength of 405 nm.

### Immunofluorescence Assay

For Immunofluorescence assay, the cells were cultured on coverslips for 24 h. And then, cells were fixed in pre‐cooled methanol for 15 min and permeabilized in PBS containing 0.5% TritonX‐100 for 10 min at room temperature. Slides were rinsed three times with PBS and blocked with 5% BSA for 30 min. Then, the slides were incubated with related antibodies at 4 °C overnight. Slides were rinsed three times with PBS, followed by exposure to Alexa‐Fluor‐488‐conjugated goat anti‐rabbit antibody and Alexa‐Fluor‐555‐conjugated goat anti‐mouse antibody (all from Invitrogen) for an additional 1 h at 37 °C. Cells were co‐stained in the dark with 4, 6‐diamidino‐2‐phenylindole (DAPI) and mounted using Fluorescence Antifade Mountant. Immunostained cells were viewed using a confocal microscope.

### Immunohistochemical Staining

For immunohistochemical staining (IHC), samples were dewaxed with xylene and rehydrated with graded ethanol. And then, the samples were boiled in citrate buffer for 15 min for antigen retrieval and incubated with 3% hydrogen peroxidase for 10 min to blunt endogenous peroxidase. Next, sections were blocked in normal goat serum for 20 min to prevent nonspecific staining and incubated with first antibodies overnight at 4 °C. After rinsing with PBST, a secondary antibody was used, followed by incubation with DAB Chromogen dilution solution. The sections were dehydrated by ethanol series, sealed with neutral resin, and photographed under a microscope. Image J was used to analyze the pictures of IHC.

### Bioinformatics Analysis

Kyoto Encyclopedia of Genes and Genomes (KEGG) pathway analysis and Gene Ontology (GO) analysis were performed by DAVID Bioinformatics (https://david.ncifcrf.gov/tools.jsp). The data of KEGG and GO were graphed using Bioinformatics (https://www.bioinformatics.com.cn/).

### Clinical Human Ovary Cancer Specimens

Twenty normal ovary tissues and twenty ovary cancer tissues were collected from Tianjin Central Hospital of Gynecology Obstetrics (Tianjin, China). All patients of these samples have agreed to approve the use of the samples for research purposes. The study protocol was approved by the Institute Research Ethics Committee at Nankai University and Tianjin Central Hospital of Gynecology Obstetrics.

### Ethics Approval and Consent to Participate

This study was carried out in accordance with the recommendations of the Requirements of the Ethical Review System of Biomedical Research Involving Human by the National Health and Family Planning Commission of China, Nankai University, and Tianjin Union Medical Center Ethics Committee, with written informed consent from all subjects (Ethical Approval No. NKUIRB2023035). Approval of use of mice and designed experiments was given by the Laboratory Animal Ethics Committee Nankai University (Ethical Approval No. 2023‐SYDWLL‐000022).

### Quantification and Statistical Analysis

GraphPad Prism 8 software was used to analyze data. Statistical details of data include error bars, *p* values, error bars and mean values ± SD. The significant difference between different groups was analyzed by unpaired two‐tailed Student's *t* test or one‐way ANOVA. Asterisk means a *p* value < 0.05 and was considered statistically significant.

## Conflict of Interest

The authors declare no conflict of interest.

## Author Contributions

F.X., H.Z., X.D., and M.T. contributed equally to this work. F.X. contributed to the drafting of the manuscript. F.X., H.Z., X.T.D., M.X.T., C.X.Y., Y.J.G., L.M.L., H.R.S., Q.L.G., J.Y.W., M.M.S., Q.J.Z., T.Y.W., T.H., Z.L., Y.P.L., and Z.J.C. were involved in the acquisition of data, data analysis and interpretation, and statistical analysis. T.W. and J.G.Z. participated in the collection of clinical samples. Z.L., J.Y.W., M.M.S., C.Z.Z., S.Z., and C.L.S. provided funding for the study. S.Z. and C.L.S. contributed to the study concept and design, as well as study supervision. C.L.S. was responsible for writing, reviewing, and editing the manuscript. All authors have read and approved the final manuscript.

## Supporting information



Supporting Information

Supporting Table 1

Supporting Table 2

Supporting Table 3

Supporting Table 4

Supporting Table 5

## Data Availability

The data that support the findings of this study are available on request from the corresponding author. The data are not publicly available due to privacy or ethical restrictions.
