# Supplementary material for: HPD is an RNA‐Binding Protein Sustaining Ovarian Cancer Cell Glycolysis, Tumor Growth, and Drug Resistance
Source: Adv Sci (Weinh). 2025 Jun 10;12(30):e03999. doi: 10.1002/advs.202503999 (PMC12376507; doi:10.1002/advs.202503999)
Supplement: Supplementary file 1 — Supporting Information [file ADVS-12-e03999-s002.docx]

Supporting Information

**HPD is an RNA-Binding Protein Sustaining Ovarian Cancer Cell Glycolysis, Tumor Growth, and Drug Resistance**

Fei Xie, Han Zhang, Xintong Dai, Mengxin Tu, Chenxi Yu, Lumeng Liu, Yajuan Guo, Huanran Sun, Qingle Gao, Jiyan Wang, Mingming Sun, Qijun Zhang, Taoyuan Wang, Tao He, Zhen Li, Yanping Li, Tao Wang, Jianguo Zhao, Zhongjie Chen, Chunze Zhang, Shuai Zhang*, Changliang Shan*


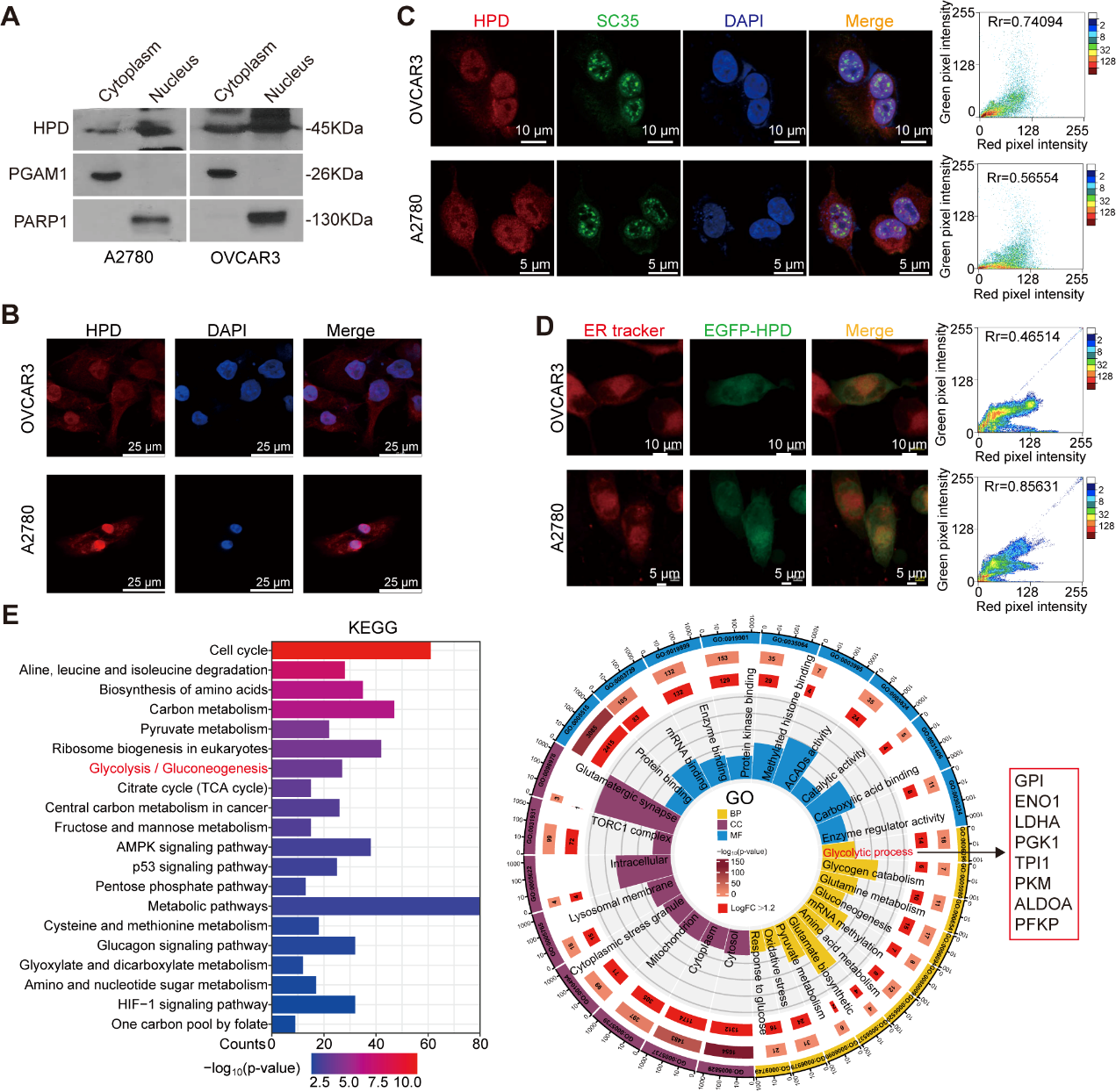


**Figure S1**

HPD is a new non-canonical RBP and associates with multiple mRNAs. A) Nucleocytoplasmic separation experiment was performed to detect the sub-cellular localization HPD. PGAM1 acted as the marker of cytoplasm, and PARP1 as the marker of nuclear. B) Immunofluorescence staining assay was conducted to detect the sub-cellular localization of HPD. C) Immunofluorescence staining assay was used to detect the co-localization of HPD and nuclear plaques in the nucleus. Nuclear speckles were recognized according to the SC35 protein. D) Immunofluorescence staining assay was used to detect the co-localization of HPD and endoplasmic reticulum in the cytoplasm. E) Kyoto Encyclopedia of Genes and Genomes (KEGG) pathway analysis and Gene Ontology (GO) analysis were performed to analyze the 5053 RNAs enriched by HPD.


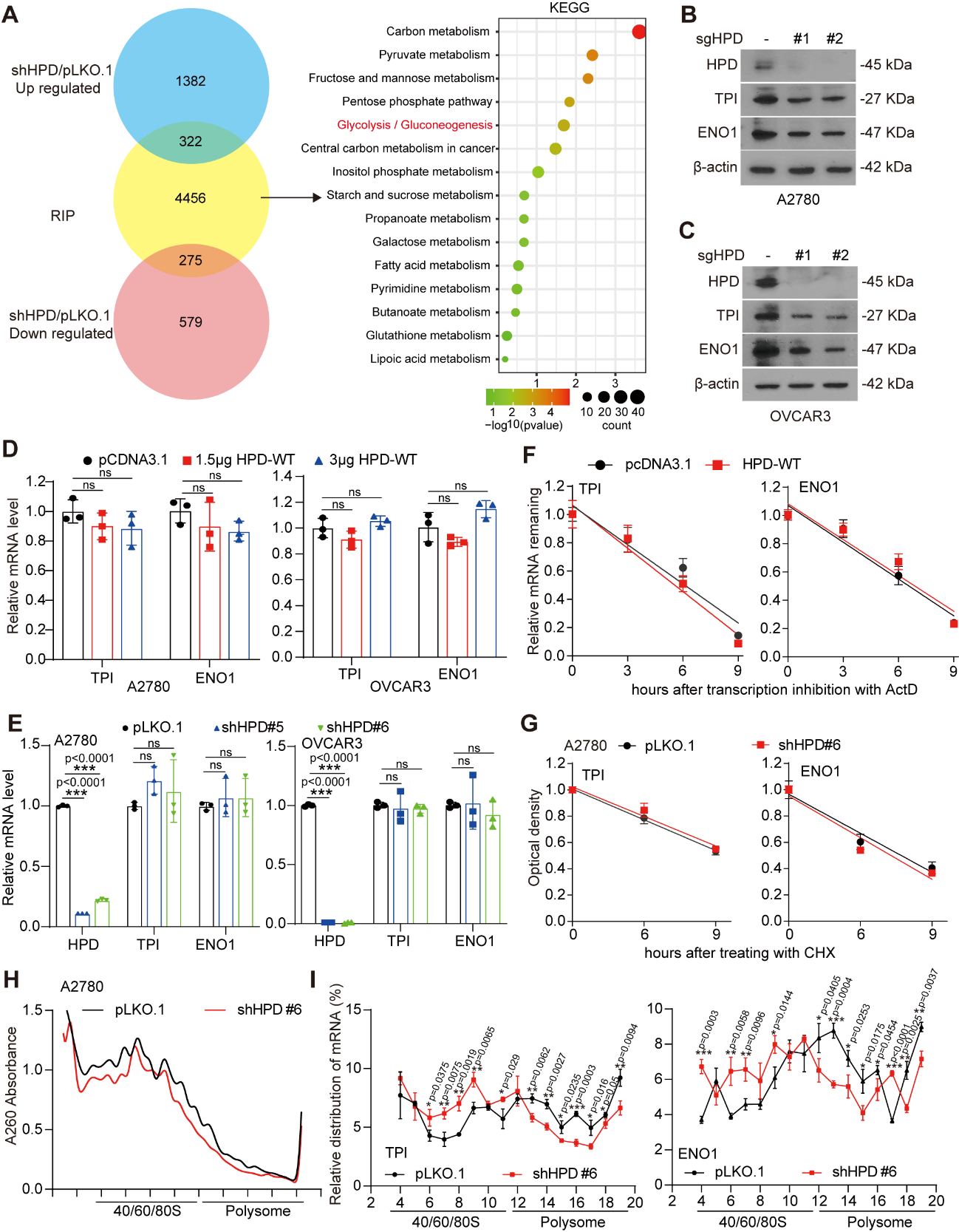


**Figure S2**

The RNA-binding activity of HPD contributes to global protein translation. A) RNA seq was performed in HPD knockdown A2780 cells. And the RNA-seq data were overlapped with the data of RIP-seq. (RNA seq, pLKO.1, n=3; shHPD, n=3). B-C) The protein expression of glycolytic enzymes was detected by western blotting in HPD knockout A2780 and OVCAR3 cells. D) The qPCR assay was performed to detect the mRNA level of TPI and ENO1 in HPD overexpressed A2780 and OVCAR3 cells. E) The mRNA levels of TPI and ENO1 in HPD knockdown A2780 and OVCAR3 cells were detected by qPCR assay. The empty pLKO.1 vector was used as negative control. F) The effect of HPD on the mRNA stability of TPI and ENO1 was detected by qPCR assay in A2780 cells treated with ActD. G) The effect of HPD on the protein stability of TPI and ENO1 were detected by western blotting in the cells treated with CHX. H) Polysome fractionation assay was performed to detect the translation level of HPD knockdown A2780 cells by sucrose density gradient centrifugation. I) Relative mRNA distribution of the TPI and ENO1 in each ribosome fractions from polysome fractionation was analyzed by qPCR assay. Error bars in D, E and I, mean values ± SD, *p* values were determined by unpaired two-tailed Student’s t test of n=3 independent biological experiments. **p* < 0.05; ***p* < 0.01; ****p* < 0.001.


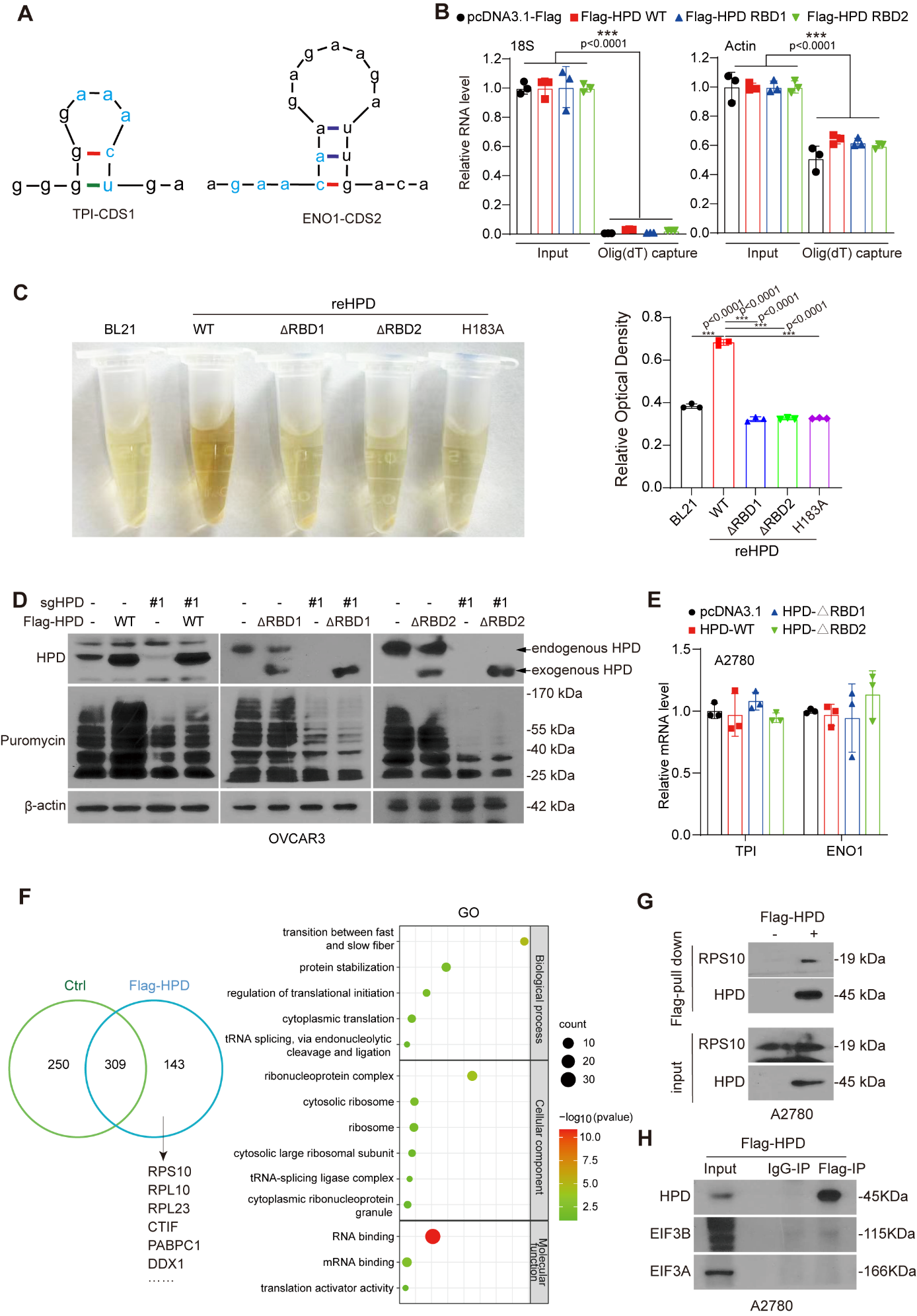


**Figure S3**

HPD binds to mRNA depending on its RBD domains. A) The structure of RNA around RRACH motif on TPI-CDS1 and ENO1-CDS2. B) Levels of 18S rRNA, β-actin and ENO1 in eluates of A2780 cells captured by oligo(dT) were evaluated by qPCR assay. C) The activity of HPD as a catalytic enzyme in the tyrosine metabolic pathway was examined *in vitro*. D) The puromycylation assay was performed to detect global translation efficiency after rescuing flag-HPD WT or truncated mutant in HPD knockout OVCAR3 cells. E) The qPCR assay were performed to detect the mRNA level of TPI and ENO1 in HPD-WT or truncated mutant overexpressed A2780 cells. F) HPD-bound proteins were identified by protein mass spectrometry, and GO analysis was performed. (Control, n=1; Flag-HPD, n=1). G) Flag-pull down was performed to detect the binding of Flag-HPD and RPS10. H) Flag-IP was performed to examine the binding of Flag-HPD and EIF3A/3B. Error bars in B and C, mean values ± SD, *p* values were determined by unpaired two-tailed Student’s t test of n=3 independent biological experiments. **p* < 0.05; ***p* < 0.01; ****p* < 0.001.


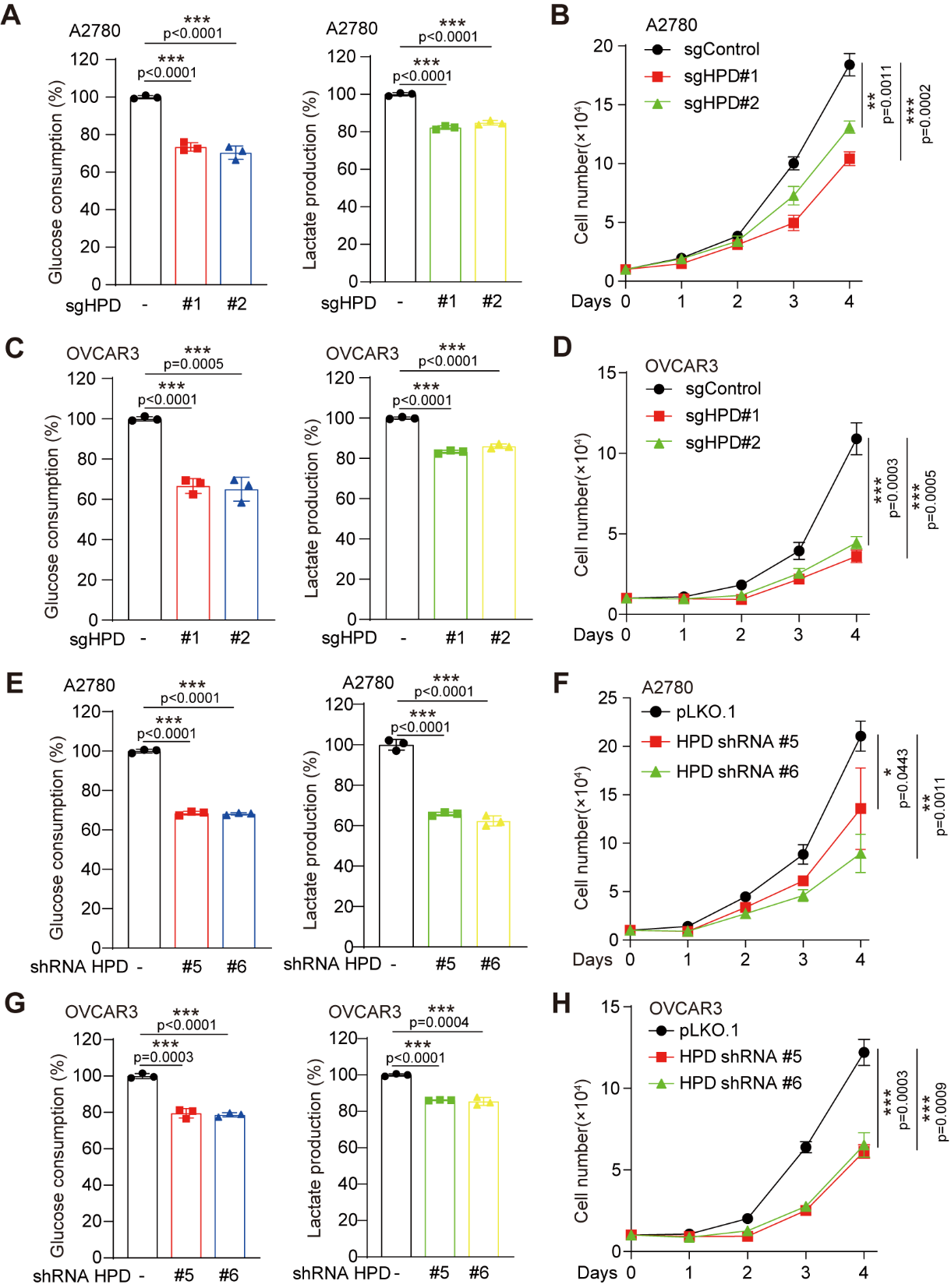


**Figure S4**

HPD promotes ovarian cancer glycolysis and tumor growth in an RBP-dependent manner. A) The relative glucose consumption and lactate production were detected in HPD knockout A2780 cells. B) The cell proliferation was determined by cell number counting in A2780 cells with HPD knockout. C) The relative glucose consumption and lactate production were detected in HPD knockout OVCAR3 cells. D) The cell proliferation was determined by cell number counting in OVCAR3 cells with HPD knockout. E) The relative glucose consumption and lactate production were detected in HPD knockdown A2780 cells. F) The cell proliferation was determined by cell number counting in A2780 cells with HPD knockdown. G) The relative glucose consumption and lactate production were detected in HPD knockdown OVCAR3 cells. H) The cell proliferation was determined by cell number counting in OVCAR3 cells with HPD knockdown. Error bars in A-H, mean values ± SD, *p* values were determined by unpaired two-tailed Student’s t test of n=3 independent biological experiments. **p* < 0.05; ***p* < 0.01; ****p* < 0.001.


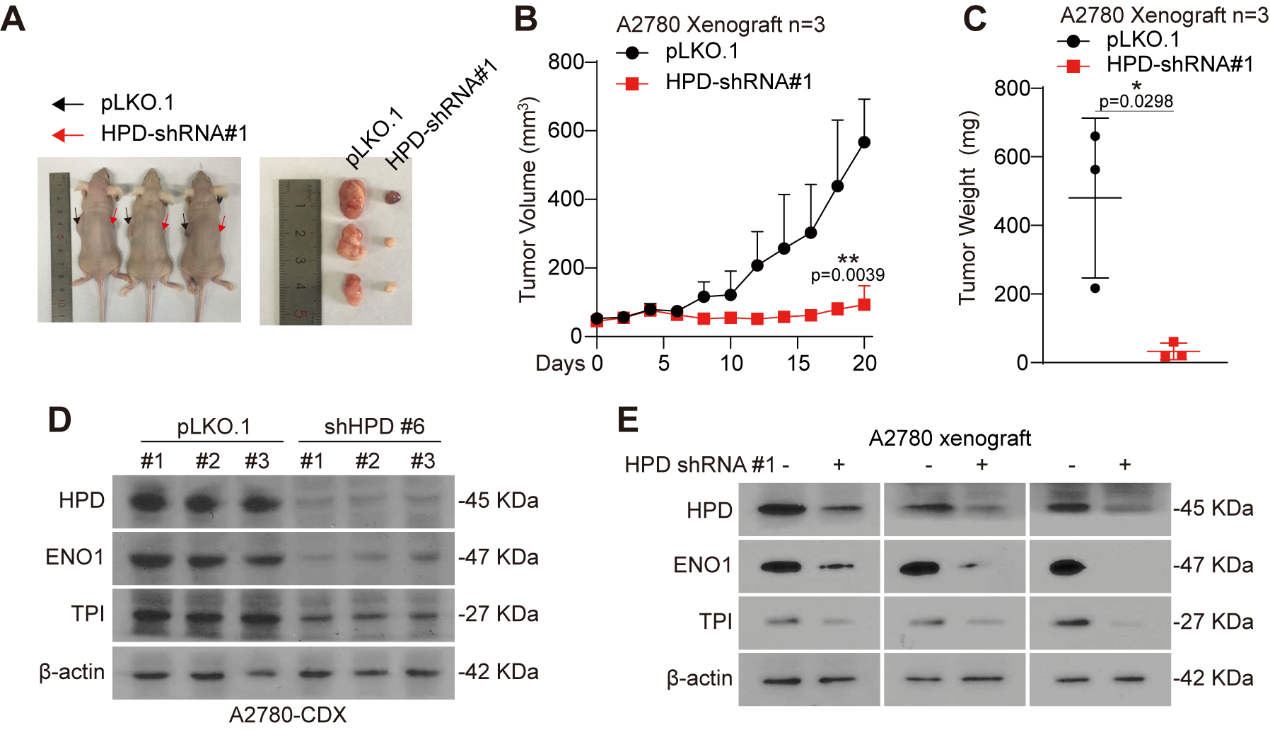


**Figure S5**

HPD promotes ovarian cancer growth. A) The tumors in xenograft nude mouse were shown. B) Tumor growth curve was recorded in xenograft nude mice bearing A2780 cells tumor with HPD knockdown. C) Tumor mass was examined in xenograft nude mice bearing A2780 cells tumor with HPD knockdown. D-E) The levels of HPD, ENO1 and TPI was detected by WB in tumor with HPD knockdown. Error bars in B and C, mean values ± SD, *p* values were determined by unpaired two-tailed Student’s t test of n=3 independent biological experiments. **p* < 0.05; ***p* < 0.01; ****p* < 0.001.


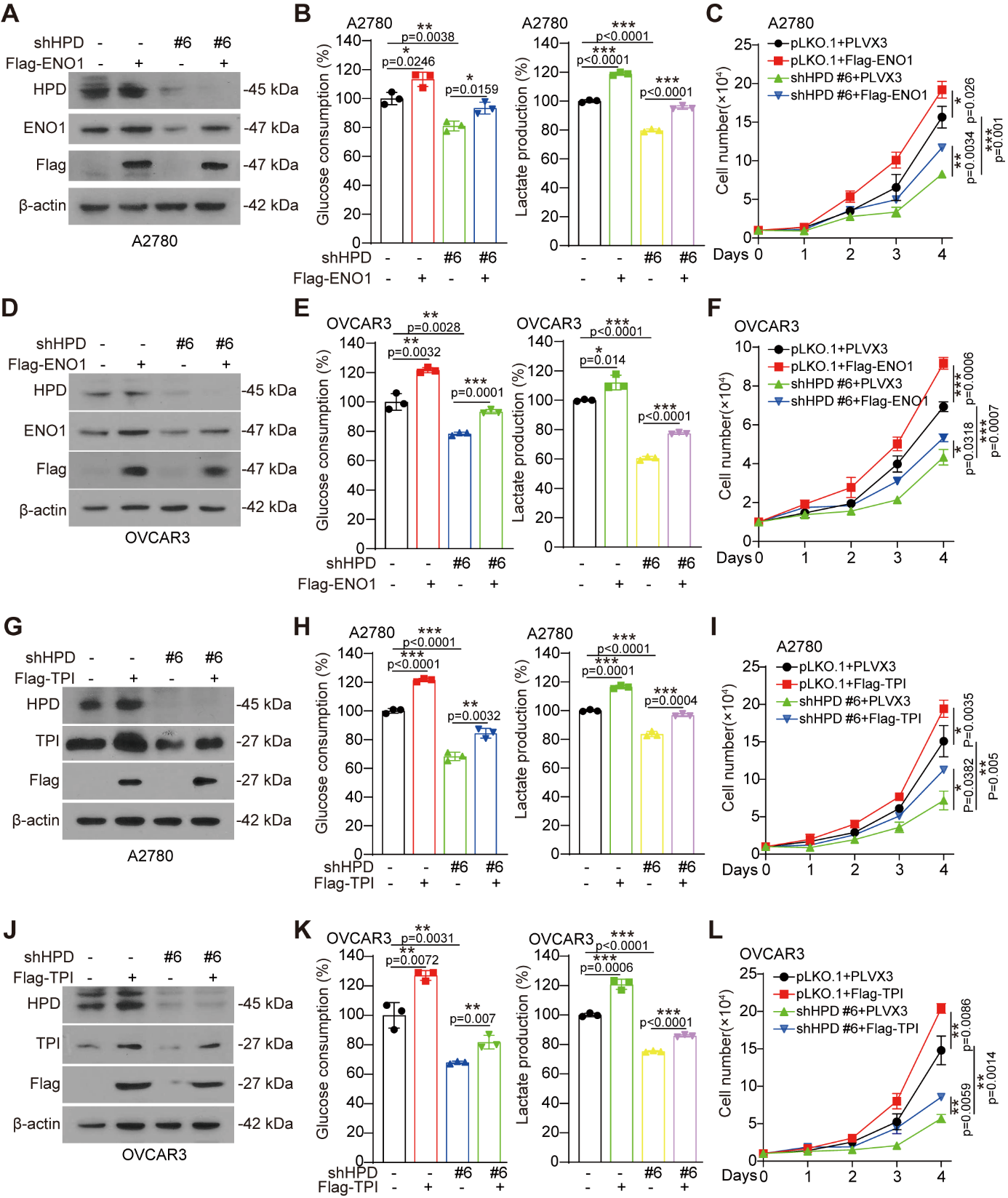


**Figure S6**

HPD promotes glycolysis and cell proliferation through ENO1 and TPI. A) The expression levels of HPD and flag were detected in HPD knockdown A2780 cells, which exogenously express with or without Flag-ENO1. B) The relative glucose consumption and lactate level were determined in A2780 cells with HPD knockdown, which exogenous express with or without Flag-ENO1. C) The cell proliferation was determined by cell number counting in A2780 cells with HPD knockdown, which exogenously express with or without Flag-ENO1. D) The expression levels of HPD and flag were detected in HPD knockdown OVCAR3 cells, which exogenously express with or without Flag-ENO1. E) The relative glucose consumption and lactate level were determined in OVCAR3 cells with HPD knockdown, which exogenous express with or without Flag-ENO1. F) The cell proliferation was determined by cell number counting in OVCAR3 cells with HPD knockdown, which exogenously express with or without Flag-ENO1. G) The expression levels of HPD and flag were detected in HPD knockdown A2780 cells, which exogenously express with or without Flag-TPI. H) The relative glucose consumption and lactate level were determined in A2780 cells with HPD knockdown, which exogenous express with or without Flag-TPI. I) The cell proliferation was determined by cell number counting in A2780 cells with HPD knockdown, which exogenously express with or without Flag-TPI. J) The expression levels of HPD and flag were detected in HPD knockdown OVCAR3 cells, which exogenously express with or without Flag-TPI. K) The relative glucose consumption and lactate level were determined in OVCAR3 cells with HPD knockdown, which exogenous express with or without Flag-TPI. L) The cell proliferation was determined by cell number counting in OVCAR3 cells with HPD knockdown, which exogenously express with or without Flag-TPI. Error bars in B, C, E, F, H, I, K and L, mean values ± SD, *p* values were determined by unpaired two-tailed Student’s t test of n=3 independent biological experiments. **p* < 0.05; ***p* < 0.01; ****p* < 0.001.


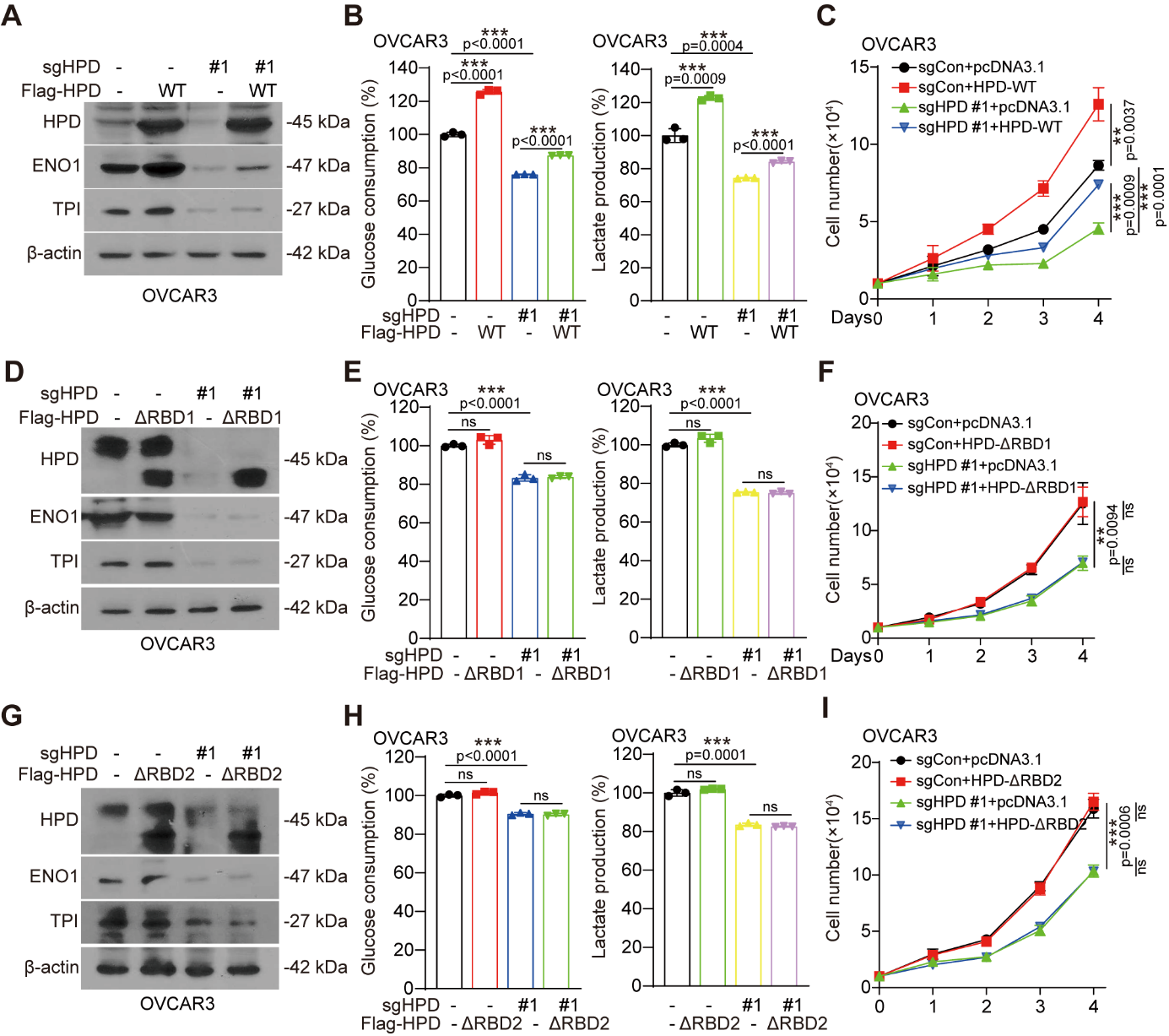


**Figure S7**

HPD promotes glycolysis and cell proliferation in a RBP-dependent manner. A) The expression levels of HPD, ENO1 and TPI were detected in HPD knockout OVCAR3 cells, which exogenous express with or without Flag-HPD-WT. B) The relative glucose consumption and lactate level was determined in OVCAR3 cells with HPD knockout, which exogenous express with or without Flag-HPD-WT. C) The cell proliferation was determined by cell number counting in OVCAR3 cells with HPD knockout, which exogenous express with or without Flag- HPD-WT. D) The expression levels of HPD, ENO1 and TPI were detected in HPD knockout OVCAR3 cells, which exogenous express with or without Flag-HPD-△RBD1. E) The relative glucose consumption and lactate level was determined in OVCAR3 cells with HPD knockout, which exogenous express with or without Flag-HPD-△RBD1. F) The cell proliferation was determined by cell number counting in OVCAR3 cells with HPD knockout, which exogenous express with or without Flag- HPD-△RBD1. G) The expression levels of HPD, ENO1 and TPI were detected in HPD knockout OVCAR3 cells, which exogenous express with or without Flag-HPD-△RBD2. H) The relative glucose consumption and lactate level was determined in OVCAR3 cells with HPD knockout, which exogenous express with or without Flag-HPD-△RBD2. I) The cell proliferation was determined by cell number counting in OVCAR3 cells with HPD knockout, which exogenous express with or without Flag- HPD-△RBD2. Error bars in B, C, E, F, H and I, mean values ± SD, *p* values were determined by unpaired two-tailed Student’s t test or One Way ANOVA of n=3 independent biological experiments. **p* < 0.05; ***p* < 0.01; ****p* < 0.001.


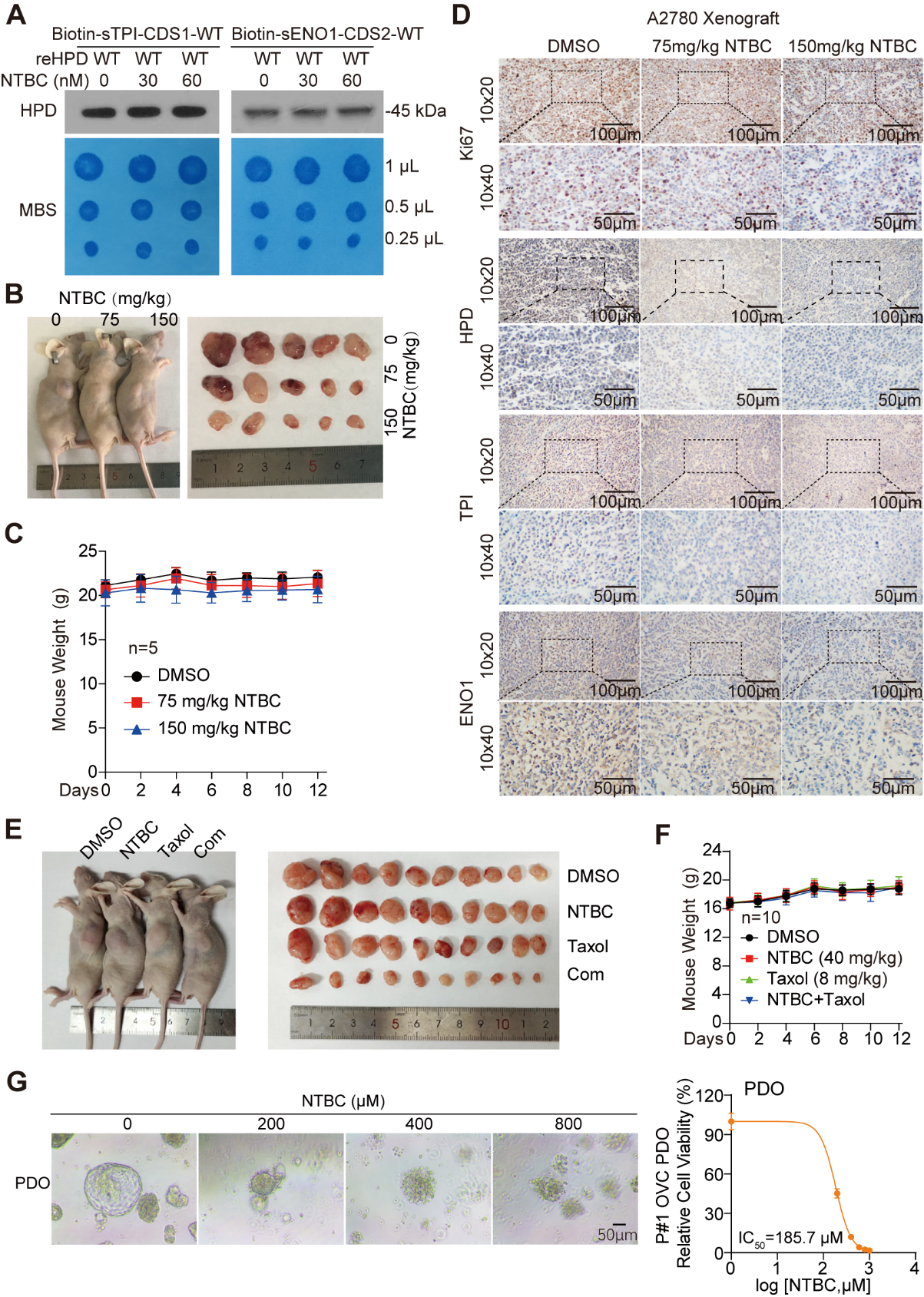


**Figure S8**

Targeting HPD RNA binding ability abrogates tumor growth and enhances Taxol response. A) HPD protein and RNA were detected after treating with NTBC by western blot and MBS (methylene blue staining), respectively. B) The tumors in xenograft nude mouse were shown. C) Body weights of mice in each group were recorded. D) The IHC was performed to detect the level of Ki67, HPD, TPI and ENO1 in the representative tumor. E) The tumors in xenograft nude mouse were shown. F) Body weights of mice in each group were recorded. G) Ovarian cancer patient-derived organoids (PDO) models treated with different concentrations of NTBC was detected.
